# A review of plasma-assisted catalytic conversion of gaseous carbon dioxide and methane into value-added platform chemicals and fuels

**DOI:** 10.1039/c8ra03146k

**Published:** 2018-08-02

**Authors:** Harinarayanan Puliyalil, Damjan Lašič Jurković, Venkata D. B. C. Dasireddy, Blaž Likozar

**Affiliations:** Department of Catalysis and Chemical Reaction Engineering, National Institute of Chemistry Hajdrihova 19 1001 Ljubljana Slovenia damjan.lasic@ki.si

## Abstract

CO_2_ and CH_4_ contribute to greenhouse gas emissions, while the production of industrial base chemicals from natural gas resources is emerging as well. Such conversion processes, however, are energy-intensive and introducing a renewable and sustainable electric activation seems optimal, at least for intermediate-scale modular operation. The review thus analyses such valorisation by plasma reactor technologies and heterogeneous catalysis application, largely into higher hydrocarbon molecules, that is ethane, ethylene, acetylene, propane, *etc.*, and organic oxygenated compounds, *i.e.* methanol, formaldehyde, formic acid and dimethyl ether. Focus is given to reaction pathway mechanisms, related to the partial oxidation steps of CH_4_ with O_2_, H_2_O and CO_2_, CO_2_ reduction with H_2_, CH_4_ or other paraffin species, and to a lesser extent, to mixtures' dry reforming to syngas. Dielectric barrier discharge, corona, spark and gliding arc sources are considered, combined with (noble) metal materials. Carbon (C), silica (SiO_2_) and alumina (Al_2_O_3_) as well as various catalytic supports are examined as precious critical raw materials (*e.g.* platinum, palladium and rhodium) or transition metal (*e.g.* manganese, iron, cobalt, nickel and copper) substrates. These are applied for turnover, such as that pertinent to reformer, (reverse) water–gas shift (WGS or RWGS) and CH_3_OH synthesis. Time-on-stream catalyst deactivation or reactivation is also overviewed from the viewpoint of individual transient moieties and their adsorption or desorption characteristics, as well as reactivity.

## Abstract 2

The review focuses on the valorisation of two major greenhouse gases (methane and carbon dioxide) utilising different hybrid plasma reactors where valuable chemicals such as higher hydrocarbons, alcohols, aldehydes, carboxylic acids, *etc.* are produced.

## Graphical abstract

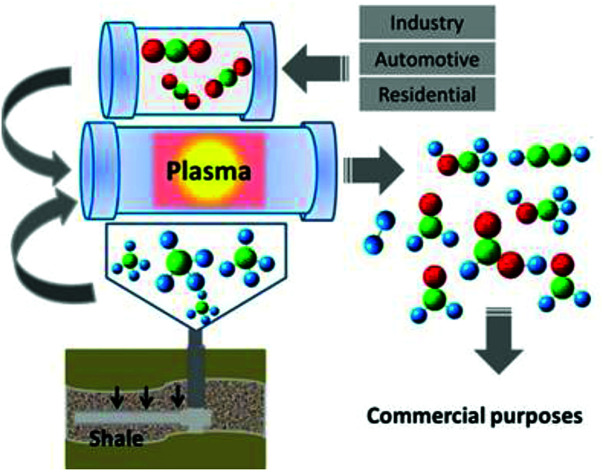

## 1. Introduction

According to some recent reports, 80% of the global energy requirements are satisfied by fossil fuels like oil, natural gas and coal.^1^ Due to its vast availability and lower emissions compared to oil or coal, natural gas consumption has increased significantly over past few decades. Natural gas reserves including both conventional and unconventional resources are estimated to be about 6330 trillion m^3^.^2^ It is demonstrated that CO_2_ emissions from natural gas combustion are 25–45% lower compared to those from coal or oil.^3^ Besides, it emits much lower quantities of toxic components including various oxides (NO_*x*_, SO_*x*_ and CO), aromatic hydrocarbons and genotoxic materials.^1,4^ Natural gas primarily contains CH_4_ (≤95%) and is conventionally obtained as a by-product of petroleum refining. The major unconventional source of natural gas is termed shale gas, where CH_4_ is trapped in the micro pores of sedimentary rocks (shales). The gas can be recovered by hydraulic fracturing combined with horizontal drilling of the shale. Challenges, potential opportunities and related environmental issues of hydraulic recovery of CH_4_ have been addressed elsewhere.^5,6^ Another potentially abundant methane source are natural gas hydrates.^7^

CH_4_ is directly used as a feedstock chemical as well as a fuel for energy production in residential, electric power, industrial and transport sectors.^8^ Liquefaction of CH_4_ is particularly important since most of the natural gas deposits are located at remote areas and long distance transportation is imperative to make it available to the market. However, compression of CH_4_ into liquid form requires considerable amounts of energy, which are not economical for commercial purposes.^9^ Due to a very low boiling point (−161.6 °C at a pressure of 1 atm) and high flammability, transportation of CH_4_ from stranded sources is challenging and its uses are limited.^10^ For this reason, research on controlled oxidation of CH_4_ to liquid fuels like CH_3_OH or conversion to easily liquefiable hydrocarbons has been given a lot of attention in the past decades.^11,12^ A new advance in the technology for the cost effective conversion of CH_4_ to liquids would allow easy storage and transportation, and exploitation of CH_4_ on a much larger scale. For instance, the urge for CH_3_OH production is increasing since CH_3_OH can be used in fuel cells for energy production.^13–15^ The major challenges that confront CH_4_ activation are the high bond dissociation energy (104 kcal mol^−1^) and covalent nature of C–H bonds. CH_4_ cannot form efficient coordinate bonds with transition metal catalysts or other electron deficient centres due to a low basicity of the molecule. As reported by Olah *et al.*, only strong super acids can generate methyl carbocation by hydride ion abstraction.^16,17^

Leakage of CH_4_ into the atmosphere due to its high volatility is a serious concern. It is estimated that around 8% of CH_4_ escapes to the atmosphere during shale gas recovery, transportation, storage and distribution.^18^ This number can be reduced if we can convert CH_4_ into less volatile or liquid forms. A report in 2010 reveals that the relative CH_4_ contribution to the greenhouse effect is the second highest (16%), preceded only by CO_2_ (76%).^19^ According to some rough estimation, CH_4_ emission into the atmosphere is expected to increase by up to 23% by 2020 (equivalent to 8 million metric tons of CO_2_). Nevertheless, both CH_4_ and CO_2_ emissions have increased drastically and have been given increased attention in past decades due to the extensive use of fossil fuels and exploitation of natural gas on a very large scale.

To control the global warming and related climate changes, conversion of CH_4_ and CO_2_ into other convenient forms is essential. The research on the catalytic conversion of CO_2_ and CH_4_ for sustainable development and carbon cycle fixation is blooming.^20–22^ In general, photochemical, electrochemical, biological, catalytic or plasma assisted conversion methods are used to convert CO_2_ into useful compounds including urea, alcohols, carboxylic acids, lactones, heterocyclic compounds and polymeric materials.^20,23–26^ Similarly, CH_4_ conversion with thermal or catalytic pyrolysis, oxidative coupling, biological processes or plasma activation techniques is efficiently used for the large scale production of valuable products including higher hydrocarbons, hydrogen, synthesis gas, alkanes, CH_3_OH, carboxylic acids, alkenes or aromatics.^27–30^ It is worth mentioning that most desired products are liquid organic oxygenates and C-5+ hydrocarbons. However, even C-2 to C-4 hydrocarbons are easily compressible compared to CH_4_, which is promising for cheaper transportation. However, some of these compounds have strictly regulated transport and storage. For example, acetylene can spontaneously explode when stored at high pressures, typically above 2 bar, which is why it is mostly transported and stored by dissolving it in acetone. Despite of such practical challenges, the conversion of CH_4_ to various chemicals (for example acetylene) is considered as a challenge for the scientific community from the perspective of C–H bond activation.

The greatest potential for a major advance in CH_4_ catalytic conversion technology is in the discovery of a route for the formation of oxygenates *i.e.*, CH_3_OH and formaldehyde.^28,31–34^ Presumably, H_2_ liberated as a by-product of the dehydrogenation of CH_4_ reacts with O_2_ on the catalyst to form a surface peroxide species, which is responsible for the activation of CH_4_ to oxygenated compounds.^31^ There are some reports for oxidation of CH_4_ using oxygen over molybdenum based catalysts, in which very low conversions of CH_4_ (<10%) and a high selectivity (50%) towards formaldehyde were observed.^35,36^ Zeolite based catalysts are also explored in CH_4_ to CH_3_OH reaction and the ability of Cu-exchanged zeolites to convert CH_4_ to CH_3_OH at 130–200 °C using molecular oxygen as the oxidant was discovered.^32,35,37^

Nevertheless, it was found that CH_4_ conversion and rates of CH_3_OH formation drastically decreased due to several deactivation mechanisms, mainly coking and sintering. To overcome this deactivation, hydrogen was used as a co-feed (at temperatures above 400 °C), but it had a detrimental effect on CH_4_ conversion and CH_3_OH selectivity. In recent studies, a Fe-based zeolite showed catalytic formation of CH_3_OH from CH_4_ in aqueous hydrogen peroxide.^37,38^ Cu–Fe/ZSM-5 catalyst was reported as an active catalyst for the oxidation of CH_4_ to CH_3_OH. However, due to the high temperatures required for the reaction, reasonable selectivity was obtained only at insignificant conversions of 0.25% due to further oxidation.^39^

Similar to CH_4_, CO_2_ is a carbon resource that can be transformed into useful chemicals such as CH_3_OH, dimethyl ether, *etc.* by thermal catalysis^40,41^ Although most of the research focuses on CO_2_ hydrogenation to CH_3_OH, CO_2_ conversions remain low (<20%) due to difficulties in activating CO_2_.^42,43^ Industrial catalysts that are effective for CO-rich feed are not as effective for CO_2_-rich feeds under similar operation conditions.^44^ It is well established, however, that copper-based catalysts are typically used for CH_3_OH synthesis (at 240–260 °C and 40–50 bar) from CO_2_.^43,45^ Also due to feedstock differences, Cu/ZnO-based catalysts have been widely investigated and modified with various metal oxides for the hydrogenation of CO_2_.^46–48^

Although some superior catalysts for CO_2_ hydrogenation have been reported, the majority of new catalysts still contains copper as the main component, together with various modifiers and supports.^43,49,50^ In addition, several detailed studies were devoted to the influence of Ga and Pd and it was found that the catalytic performance of the Cu/Zn-based catalyst was improved when even little amounts of Ga and Pd were added.^51,52^ Nevertheless, the high cost of these additives hindered their practical application. However, the CH_3_OH yield in most experiments is typically below 30%. In addition to this, the hydrogenation of CO_2_ to CH_3_OH process requires a high quantity of H_2_ and has low CH_3_OH selectivity. Thus, CH_3_OH production using this method is still far from an economical point of view.^41^

As addressed before, catalytic conversion of CH_4_ and CO_2_ is associated with numerous issues such as high temperature operation, coking, sintering and low conversion rate and product selectivity. A big limitation of any thermal activation reaction of CH_4_ to liquid products is that most such products start decomposing at temperatures required for meaningful CH_4_ conversion, which makes it practically impossible to achieve high conversions and selectivities simultaneously, preventing high single-pass product yields. Recent developments in the field of plasma assisted conversion techniques provide a partial or complete solution to many of these issues. For this reason, considerable attention has been given to the research in the field of plasma assisted valorisation of CH_4_ and CO_2_.^53–57^ Inside the plasma, electron impact excitation, dissociation and ionization can generate numerous species including excited molecules, neutral atoms, atomic or molecular ions and metastable species at room temperature.^58,59^ Thus generated reactive species undergo recombination reactions to yield neutral molecules with upgraded product values.

Product formation inside plasma can be controlled by changing the density of the species generated in the system. This can be done with the regulation of various parameters such as gas flow rate, discharge power and feed gas composition. In many cases, by introducing suitable catalysts inside the discharge zone, the yield and selectivity of the product formation are improved.^60^ One of the roles of the catalyst inside plasma is to provide a suitable surface for efficient adsorption of various reactive species, which can modify the reaction pathways. In addition, the catalyst in the discharge zone can significantly modify the properties of the generated plasma by inducing effects such as surface discharge, improving the ionization and dissociation processes and many more. These influences are highly dependent on the physical and chemical characteristics of the packing material. The effects of catalyst on properties of plasma and *vice versa* are discussed in detail in the following sections. In this review, current state of art in the field of plasma assisted catalytic and non-catalytic processes used for CH_4_ and CO_2_ activation and conversion into valuable chemicals (especially higher hydrocarbons and organic oxygenates) and underlying mechanisms are provided. Important surface processes and role of plasma in both physical and chemical modification of various catalyst materials are also discussed.

## 2. Plasma: the fourth state of matter

Plasma is a state of matter that is essentially an ionized gas. Approximately 99% of our universe consists of plasma. An unambiguous definition of plasma cannot be easily determined, and plasma is usually defined as such when gas has certain properties, such as electrical conductivity, light emission, high gas dissociation levels *etc.* In plasma state, the gas contains a wide spectrum of different species like ions, electrons, atoms, radicals and neutral molecules, as well as their excited forms. Plasma is not charged overall; however, it contains free charge carriers and is therefore electrically conductive.^61^

### 2.1 Plasma generation

Plasma can be generated from neutral gas by different processes; thermal excitation being the most intuitive one. When gas gets heated to a high enough temperature, which is usually on the order of thousands of degrees, gas molecules have a high enough energy for spontaneous dissociation, excitation and ionization to take place. Thus, plasma is essentially formed because it's thermodynamically more favourable for the gas to be dissociated or ionized at that temperature. However, this methodology for plasma generation is not applauded due to high energy consumption and other technical issues associated with the operation.

The second, kinetically more interesting and widely accepted approach for plasma generation is generation by means of electricity. A typical example is capacitively coupled plasma, where a high potential difference is applied between two electrodes. Due to the effects of electric field which causes an electrical discharge, the gas in between the electrodes is transformed into plasma.

The way discharge plasma generation works is that with a high enough voltage, termed breakdown voltage, current will start to flow through the otherwise non-conductive gas. The voltage can be on the order of thousands of volts, and for gas at atmospheric pressure, it can be very roughly estimated to about 3 kV for a 1 mm distance of gas between electrodes.^62^ Therefore, the strength of electric field in the gas is of a very high magnitude. This causes the electrons to have a high acceleration rate going from one electrode towards the other, and they can develop very high energies. Generally, the electrons will be accelerated until they interact with another particle, like a gas molecule. Therefore, at lower pressures, it is expected that the electrons will develop higher energies, because the mean free collision path with molecules is longer.

Accelerated electrons interact with atoms and molecules in two different ways, the first being so-called elastic collisions. In this type of interaction, electrons will transfer some of their energy to the molecule in form of kinetic energy, *e.g.* heat. This is because the energy transfer during the collision is not sufficient to cause any chemical changes in the molecule and just raises its temperature. The other type of interactions is termed inelastic collisions. In this case, when an electron of appropriate energy collides with an atom or molecule, the electron energy transfer is sufficient to induce phenomena such as excitation, ionization or dissociation. This generally supplies significantly less heat to the system, as most of the electron energy is used for state changing in order to overcome some of the associated energy barriers.

Both low pressure and atmospheric pressure plasmas have proved their vital roles in various fields of research including physics, chemistry, biology, medicine and material science.^63–68^ However, in chemical conversion routes, operational pressure close to atmospheric pressure is vastly accepted. The reason for this is that using low pressure is both expensive and harder to manage, and is also not favourable for reactions from a kinetic approach. Indeed, high pressure is generally used in those industrial catalytic processes where the thermodynamics doesn't restrict it, to speed up the reaction. While it's possible to use plasma at higher pressures, it is usually not preferred because increasingly high voltages have to be used for the discharge. The voltage limitations may be overcome by reducing the discharge gap. However, the gap might be too narrow for any potential catalyst incorporation in the discharge zone. Therefore, it comes to no surprise that in CO_2_ and CH_4_ activation chemistry, most of the research is done using atmospheric pressure plasmas.

The setups to generate discharge plasma are many. The ones mainly used in CH_4_ and CO_2_ valorisation processes are briefly described below. The first obvious example is a spark plasma reactor, in which, two electrodes are placed at a distance, usually on the mm-cm order, and then AC voltage is applied to one of the electrodes, forming “sparks” between them. The electrode can be either narrow in shape, or one of them can be wider. Narrow electrodes produce a linear, spark-like plasma volume, whether the latter setup forms plasma in a conical shape.^69^

Another common setup for plasma generation is the gliding arc reactor, in which the electrodes are placed at an angle, so the distance between them increases in the flow direction of the gas. An arc is ignited at the shortest distance between electrodes, and then travels with the gas until it dies out because it cannot be sustained anymore at increased distance. For more information on gliding arc and their applications, the reader is referred to.^62^ Another of widely used plasma setup is rotating arc or vortex flow gliding arc, which is inherently very similar. It's different in a way that it has 3D geometry using a tube an inner cone as the electrode. Gas is introduced in a swirling motion and the reactor is capable of producing plasma on a larger volume.^70^

Dielectric barrier discharge (DBD) is a somewhat different plasma setup. It differs from the ones mentioned above in the fact that it has a layer of dielectric material between the gas and the electrodes. This dielectric material can pass a limited small amount of current in one direction, called the displacement current. However, it can conduct AC electricity. When the electricity is applied, the charge accumulation on the dielectric material can generate plasma as short lived streamers. The main advantage of using a dielectric in the way of the discharge is that it will stop the discharge on one spot after a streamer is formed, and thus forcing the discharge to happen on another spot on its surface, so plasma is generated as short lived streamers.^71^ This assures that the discharge forms all over the dielectric/electrode surface. On the other hand, in the reactor types discussed above, plasma is sustained in a single channel. Characteristics of various plasma systems mentioned above are presented in Table 1. Note that only the reactor setups used for CH_4_ and CO_2_ valorisation process are shown here, and arc plasma is added for comparison as it's a typical example of thermal plasma. Therefore, some widely used plasma generation systems such as microwave plasma or glow discharge have been excluded. Another important parameter is whether a pulsed plasma source is used, which is essentially a very fast turn-on/off switch.

**Table 1:** Properties of plasma in different reactor types. Table partially reconstructed from ref. 53 and 64–69

Reactor type	DBD	Spark	Corona	Gliding arc	Arc
Scheme	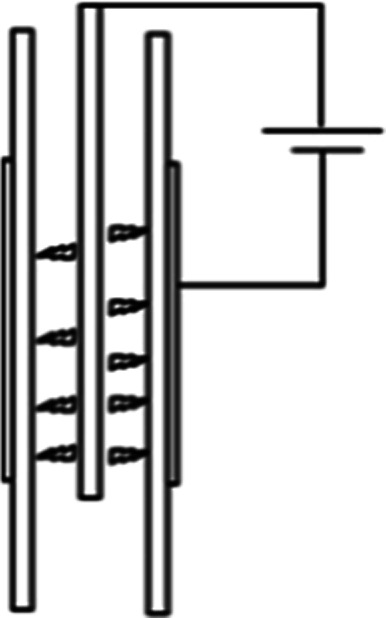	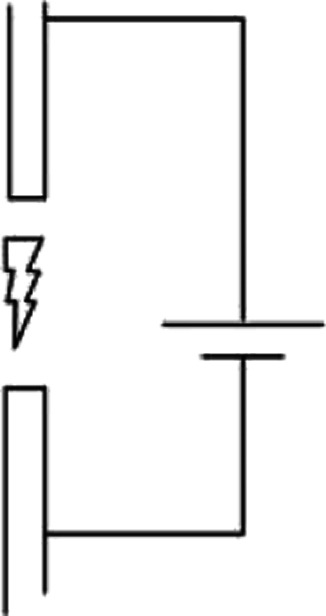	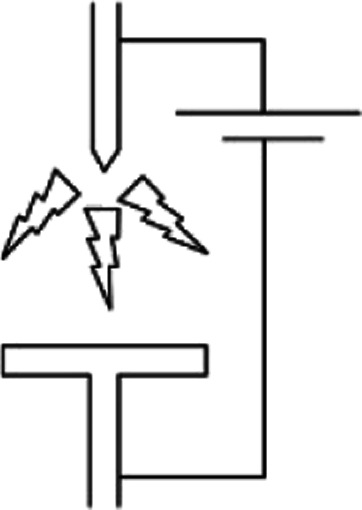	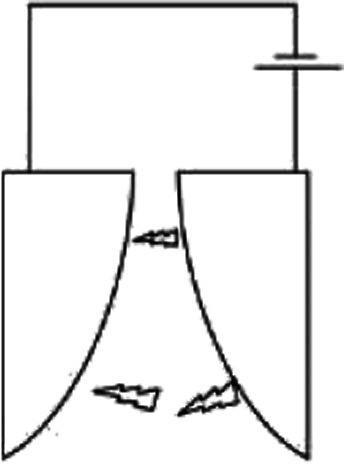	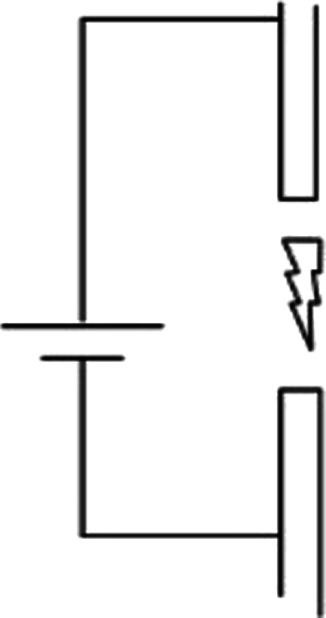
Electron energy [eV]	1–30	—	∼5	1.4–2.1	1–10
Electron density [cm^−3^]	10^12^–10^15^	10^14^–10^15^	10^9^–10^13^	10^14^–10^15^	10^15^–10^19^
Current [A]	1–50	20–30	∼10^−5^	0.1–50	30–30 000
Gas temperature [K]	300–500	400–1000	∼400	1000–3000	5 × 10^3^–10^4^
Breakdown voltage [kV]	5–25	5–15	10–50	0.5–4	10–100

The above mentioned setups, along with different parameters such as current, frequency, discharge gaps and voltage, can give a vast variety of plasma properties. One of the most important of these properties is the warmness of the plasma, which can be explained as a characteristic depending on the type of electron collision interactions in specific conditions. More details on warm and cold plasmas are illustrated in the following section.

### 2.2 Thermal and non-thermal discharge plasmas

The aforementioned electron collision types affect greatly the thermal properties of the plasma in question. Regarding those properties, we usually divide plasmas into thermal and non-thermal (cold) ones. Generally, in thermal plasmas, the gas temperature is relatively high and is comparable to electron temperature – the electrons and other species are in thermodynamic equilibrium. Non-thermal plasma temperature can be very low, even around room temperature, but it still contains high temperature electrons and excited and ionized species.

The two main parameters that mostly affect the “warmness” of the plasma are electron density and pressure. Plasmas with high electron density, such as arc plasma, will be very warm due to the increased amount of elastic collisions, which heat up the gas. High electron density mainly increases the temperature because the energy input per volume is higher. In setups such as DBD, the electron density is low, and the plasma is relatively cold. Table 1 presented above lists some common plasma setups with accompanying characteristics.

It should be noted that all plasma sources discussed in the paper are inherently non-thermal. However, they can still differ greatly in the gas temperature, and are often referred to as cold and warm plasmas, to signify this difference. Herein, we consider DBD and corona as cold plasmas, and other systems such as spark and gliding arc as warm plasmas.

## 3. Plasma as a reaction medium

High concentrations and potential energies of radicals, ions and excited species in plasma mean that many reactions between said species will occur. Therefore, plasma is a highly reactive medium and exhibits a high potential for many chemical processes. Non-thermal plasma seems especially promising because of its inherent low temperature, which might be favourable for certain processes due to thermodynamics. An example of such a reaction is formation of organic oxygenates, that might be unstable at high temperatures, especially where oxidants are present in the system, as in the case of CO_2_ and CH_4_ reactions to liquid products. Therefore, plasma seems like a potential candidate for the conversion of CH_4_ and CO_2_ to liquid chemicals, fuels, and other value-added chemicals. In the following sections, a more detailed insight into plasma chemistry of CH_4_ and CO_2_ is given.

### 3.1 Activation of CH_4_ and CO_2_ in plasma

CH_4_ and CO_2_ are generally considered to be relatively stable molecules that are hard to activate at low temperatures. Therefore, in such processes where these two molecules have to convert to useful products, high temperatures or aggressive co-reagents are required, as stated in the introduction section. In plasma, CH_4_ and CO_2_ can be activated or dissociated by means of high energy electron impact, which can be achieved even at room temperature. In this section, the mechanisms of CH_4_ and CO_2_ activation in plasma are discussed, whereas the mechanistic routes for the recombination of various activated plasma reactive species to valuable products are focused in the following sections. Some of the very elementary reactions and associated electron energies are presented in Table 2. Since H_2_, O_2_ and H_2_O are also used as co-reagents in the plasma valorisation of CH_4_ and CO_2_ that some of the elementary reactions for the excitation of these molecules are also presented in the table. More details on plasma excitation mechanisms of the above mentioned molecules can be found elsewhere.^72,73^

**Table 2:** Different electron collision reactions of CO_2_, CH_4_ and various common reagents, along with their required electron energies

Reagents	Products	Interaction type	Electron energy required [eV]
CH_4_ + e	CH_3_˙ + H + e	Dissociation	8.8 (ref. 72), 9 (ref. 64)
CH_4_ + e	CH_2_˙+ H_2_ + e	Dissociation	9.4 (ref. 72), 10 (ref. 64)
CH_4_ + e	CH˙ + H_2_ + H + e	Dissociation	12.5 (ref. 72), 11 (ref. 64)
CH_4_ + e	C˙ + 2H_2_ + e	Dissociation	14.0 (ref. 72), 12 (ref. 64)
CH_4_ + e	CH_4_^+^ + 2e	Ionization	12.6 (ref. 72)
CO_2_ + e	CO + O + e	Dissociation	5.5 (ref. 70 and 71)
H_2_O + e	OH + O + e	Dissociation	5.1 (ref. 73), 7 (ref. 74)
H_2_ + e	H + H + e	Dissociation	4.5 (ref. 70)
O_2_ + e	O + O + e	Dissociation	5.1 (ref. 70)
O_2_ + e	O_2_^+^ + 2e	Ionization	12.5 (ref. 70)

It can be seen from the Table 2 that CH_4_ gets mostly activated by dissociation, where one or more hydrogen atoms get removed.^74–77^ Higher electron energies are required for the removal of more than one hydrogen, so the extent to which each of these processes will take place is heavily dependent on electron energy distribution in a given system.^76,77^ In typical non-equilibrium plasmas, electron energies on the order of 5–10 eV are expected, as previously presented in Table 1. It can be seen that as CH_4_ has a high dissociation energy (9 eV), most of the dissociation will produce CH_3_ radicals rather than CH_2_ and CH ones. It is also evident that typical oxidants used alongside CH_4_ have much lower dissociation energy (5.5 eV for CO_2_ and 5.1 eV for O_2_). Therefore, it is possible to deduce that a significant portion of CH_4_ can get activated by excited oxidant species rather than dissociation. It should be noted that other activation mechanisms can occur in plasma, such as vibrational excitation, which can cause chemical reactions as well. For example, these kinds of mechanisms are highly pronounced in microwave plasmas. However, most of the processes dealing with CO_2_ and CH_4_ conversions to liquid chemicals use other plasma sources where dissociation and ionization are indeed the main activation mechanisms.

Besides electron collision, neutral molecules can be activated in other manners. One is thermal activation, which we can expect in warm plasmas, but is in most cases negligible in cold plasmas. By thermal activation, for example, CH_4_ can gradually lose each of its hydrogen atoms. Note that relatively high temperatures are required for this reaction, usually above 300 °C with a catalyst^78,79^ and 600 °C or above without one.^80^ However, even at those temperatures the conversions are negligible, so often temperatures above 1000 °C are used.^81^ In plasma, the activation can also proceed through phenomena such as charge or radical transfer, energy transfer, excitation by another molecule *etc.* These kinds of reactions play a very important role in many reaction pathways, and can be studied separately from electron collision process in afterglow plasma.^82^

Some of these previously mentioned activated species need to react among each other in order to form products. Obviously, even more reaction pathways are possible in this case. Possible reactions include different types of interactions, recombination being the most obvious. In this process, two species like radicals react to form a stable product. In general, two highly energetic species cannot recombine in an exothermic manner, because the resulting product would be unstable at the elevated temperature. In such cases, it is necessary to remove excess energy into the environment, *e.g.* to a passer-by spectator molecule, usually a neutral molecule. These kinds of reactions can therefore only take place in so called three body collisions.

## 4. Plasma valorisation of CH_4_ and CO_2_ into valuable chemicals

Conversion of the aforementioned greenhouse gases in the presence of plasma is extensively studied. Valorisation of CH_4_ is achieved mainly by plasma non-oxidative coupling to higher hydrocarbons and oxidative coupling to organic oxygenates. In the case of CO_2_, valorisation process involves partial or complete reduction with reducing agents such as H_2_ or CH_4_. This section provides an outlook into plasma valorisation of CH_4_ and CO_2_ and the influential factors associated with it. The results from the previous reports on CH_4_ and CO_2_ valorisation in various plasma reactors are summarised in Table 3.

**Table 3:** CH_4_ and CO_2_ valorisation in various non-catalytic plasma reactors. The table is divided into three subsections based on the chemistry of the process

Feed gas ratio	Reactor setup	SEI [kJ mol^−1^]	Plasma temperature [K]	Conversion [%]	Products (selectivities [%])	Reference
**Non-oxidative methane coupling**						
5% CH_4_, 95% N_2_	DBD AC	1094.4	500	CH_4_ – 14.8%	C_2_H_6_ (10.6%)	77
					C_2_H_4_ (0.7%)	
					C_2_H_2_ (0.8%)	
					C-3 (2%)	
5% CH_4_, 95% N_2_	DBD pulsed	224.64	500	CH_4_ – 12.4%	C_2_H_6_ (6.5%)	77
					C_2_H_4_ (0.3%)	
					C_2_H_2_ (0.3%)	
					C-3 (0.9%)	
5% CH_4_, 95% N_2_	Spark AC	172.8	1000	CH_4_ – 49.4%	C_2_H_4_ (0.3%)	77
					C_2_H_2_ (86%)	
5% CH_4_, 95% N_2_	Spark pulsed	322.56	1000	CH_4_ – 83.0%	C_2_H_6_ (2.6%)	77
					C_2_H_4_ (3.1%)	
					C_2_H_2_ (45.9%)	
					C-3 (0.7%)	
5% CH_4_, 95% N_2_	Rotating arc	61.92	1000	CH_4_ – 25.8%	C_2_H_6_ (0.2%)	77
					C_2_H_4_ (1.1%)	
					C_2_H_2_ (42%)	
5% CH_4_, 95% N_2_	Gliding arc	72	3000	CH_4_ – 23.7%	C_2_H_2_ (27.2%)	77
5% CH_4_, 95% N_2_	Hollow cathode	28.8	2000	CH_4_ – 42.2%	C_2_H_6_ (1.4%)	77
					C_2_H_4_ (1.4%)	
					C_2_H_2_ (27%)	
					C-3 (0.3%)	
21.2% CH_4_, 78.8% Ar	Gliding arc, 80 mm length	2090	—	CH_4_ – 43.4%	C-2 (87.2%)	87
100% CH_4_	Gliding arc, 20 kHz, 150 mm length	273.6	—	CH_4_ – 47%	C_2_H_2_ (22%)	88
15% CH_4_, 85% Ar	Gliding arc, 20 kHz, 150 mm length	165.6	—	CH_4_ – 65%	C_2_H_2_ (7%)	88
100% CH_4_	DBD, 75 kHz, 1 mm gap, 40 mm length	867	—	CH_4_ – 18%	C_2_H_6_ (30%)	90
					C_2_H_4_ (3%)	
					C_2_H_2_ (3%)	
					C-3 to C-5 (27%)	
100% CH_4_	Spark, 5 mm gap, 50 Hz DC, 5 kV, pulsed	1059	440	CH_4_ – 65%	C_2_H_4_ (5%)	90
					C_2_H_2_ (75%)	
					C-3 to C-5 (5%)	
100% CH_4_	Microwave, 1 kHz pulses of 60 μs, 30 mbar	963.5	1500–2500	CH_4_ – 90%	C_2_H_2_ (80%)	93
100% CH_4_	Corona, 1–2 kHz	3854.1	—	CH_4_ – 72%	C_2_H_2_ (56%)	94
					C_4_H_2_ (8%)	
					C_2_H_4_ (3%)	
100% CH_4_	DBD	4624.9	—	CH_4_ – 38%	C_4_H_10_ (5%)	94
					C_2_H_2_ (4%)	
					C_2_H_6_ (25%)	
					C_3_H_8_ (10%)	
50% He, 50% CH_4_	DBD, 1.2 mm gap, 120 mm length, 3 kHz	10 350	∼373	CH_4_ – 18.4%	C_2_H_6_ (80.7%)	95
					C_2_H_4_ (6.3%)	
					C_2_H_2_ (1.3%)	
					C_3_H_8_ (5.3%)	
					C-4+ (6.5%)	
10% CH_4_, 90% Ar	DBD, 3 mm gap, 4 mL volume, 10 kHz, 3–6 kV	60	—	CH_4_ – 13%	—	76
10% CH_4_, 90% Kr	DBD, 3 mm gap, 4 mL volume, 10 kHz, 3–6 kV	68.57	—	CH_4_ – 23%	C_2_H_6_ (32%)	247
					C_2_H_4_ (4%)	
					C_2_H_2_ (4%)	
100% CH_4_	DBD, 8.8 mL volume, 10 kV, 20 kHz	—	—	CH_4_ – 55.0%	C_2_H_6_ (20.89%)	97
					C_2_H_6_ (2.01%)	
					C_3_H_6_ (12.4%)	
					C-4 (11.54%)	
					C_2_H_2_ (4.85%)	
100% CH_4_	DBD, 20 kHz, 3 mm gap, 13.6 mL volume, 40 kV, 20–50 kHz	1296	—	CH_4_ – 25.2%	C_3_H_8_ (4%)	248
					C_2_H_2_ + C_2_H_4_ (12%)	
					C_4_H_10_ (19%)	
					C_2_H_6_ (34%)	
100% CH_4_	DBD, 0.4 mm gap, 200 mm length, 6.4–8.6 kV	3342	448 (wall)	CH_4_ – 25.1%	C-2 and C-3 (80.27%)	98
100% CH_4_	DBD, electrode with disks 5 mm apart	7372.8	—	CH_4_ – 10.2%	C_2_H_6_ (45%)	99
					C_3_H_8_ (20%)	
					C_2_H_4_ (3%)	
					C_2_H_2_ (3%)	
					C-4 (10%)	
					C-5+ (12%)	

**Methane partial oxidation with O**_**2**_ **, N**_**2**_ **O or H**_**2**_ **O**						
20% O_2_, 80% CH_4_	DBD, 1 mm gap, 50 mL volume, 20 kV, 30 kHz, 2 bar	530	353 (wall)	CH_4_ – 15%	CH_3_OH (22%)	108
50% H_2_, 50% O_2_	DBD, double dielectric barrier	633.6	—	O_2_ – 90.8%	H_2_O_2_ (32.2%)	112
				CH_4_ – 66.4%	H_2_O (18.5%)	
50% Ar, 42.5% CH_4_ 7.5% O_2_	DBD, 3.5 mm gap, 17.3 mL volume, 10 ns pulses, 440 Hz, 25 kV	112	—	CH_4_ – 30%	CH_3_OH (18%)	113
				O_2_ – 96%	HCHO (2%)	
					C-2 (20%)	
5% CH_4_, 5% N_2_O, 90% Ar	DBD, 2 mL volume, 1 mm gap	1029	330 (wall)	CH_4_ – 32.2%	CH_3_OH (10%)	114
				N_2_O – 53.8%	HCHO (25%)	
					C_2_H_6_ (10%)	
75% CH_4_, 25% O_2_	DBD, 4 mm, 688 cm^2^ electrode surface	849.6	301 (cooling fluid)	CH_4_ – 24%	CH_3_OH (17%)	115
				O_2_ – 74%	Methyl formate (5%)	
					HCOOH (16%)	
					HCHO (13%)	
					C_2_H_5_OH (1%)	
80% N_2_, 10% CH_4_, 10% O_2_	DBD, cooled, 1 mm ID, twisted metallic electrode, 75 kHz	672	298 (cooling fluid)	CH_4_ – 45%	CH_3_OH (17%)	117
				O_2_ – 83%	HCHO (3%)	
					HCOOH (9%)	
50%CH_4_, 50% O_2_	DBD, cooled, 1.5 mm ID, twisted metallic electrode, 10 kHz	—	283 (cooling fluid)	CH_4_ – 12%	CH_3_OH (10%)	118
					HCHO (15%)	
					HCOOH (14%)	
50% CH_4_, 50% air	DBD, 10 kV, 10 kHz, 0.5 mm gap, 600 mm winding spiral ground	864	—	CH_4_ – 30%	CH_3_OH (9%)	107
16% CH_4_, 84% H_2_O	Capacitively coupled plasma, DC, 133–1333 Pa	345.6	—	CH_4_ – 5%	CH_3_OH (20%)	121
					HCHO (6%)	
					C_2_H_6_ (19%)	
50% CH_4_, 50% H_2_O	DBD, 2–3 kV, 250–2000 Hz, 1.8 mm ID, 500 Hz pulses of 400 ns	246.4	—	CH_4_ – 10%	CH_3_OH (7.5%)	122
				H_2_O – 5%		

**CO**_**2**_ **activation with H**_**2**_ **, CH**_**4**_ **or H**_**2**_ **O**						
67.4% CH_4_, 32.6% CO_2_	DBD, 1.8 mm gap	3600	338 (cooling fluid)	CH_4_ – 35%	Alcohols (5%)	133
				CO_2_ – 20%	Acids (5%)	
					C_2_H_6_ (19%)	
					C_3_H_8_ (9.3%)	
					C-4+ (9%)	
67.4% CH_4_, 32.6% CO_2_	DBD, 1.1 mm gap, electrode with spacing	3600	338 (cooling fluid)	CH_4_ – 55%	Alcohols (3%)	133
				CO_2_ – 37%	Acids (8%)	
					C_2_H_6_ (14%)	
					C_3_H_8_ (7.5%)	
					C-4+ (8%)	
66.8% CH_4_, 33.2% CO_2_	DBD, 1 mm gap, 200 mm length, 25 kHz	2400	333 (thermocouple in plasma)	CH_4_ – 64.3%	CH_3_COOH (5.2%)	128
				CO_2_ – 43.1%	Propanoic acid (1%)	
					CH_3_OH (0.3%)	
					C_2_H_5_OH (1.8%)	
50% CO_2_, 50% H_2_	Surface discharge, 11 kV, 7 kHz	518.4	—	CO_2_ – 15%	DME (5%)	134
50% CO_2_, 50% H_2_O	Negative corona plasma, 15 kV, 10.2 kHz	6652.8	378 (thermostat)	CO_2_ – 18%	CH_3_OH (21%)	136
				H_2_O – 14%	C_2_H_5_OH (13%)	

### 4.1 Non-oxidative CH_4_ conversion

The simplest and most obvious CH_4_ plasma valorisation technique is the so-called non-oxidative CH_4_ conversion. In the process, pure CH_4_ is fed into a plasma zone, where reaction takes place. Two main mechanism are prevalent here, activation by electron collision and thermal activation. In both mechanisms, hydrogen is stripped from CH_4_ resulting in activated species which can react with another one to form stable products. As described by the studies mentioned later in the review, main products of this process are C_2_H_6_ and C_2_H_2_ with accompanying hydrogen. Higher hydrocarbons are also formed, but are increasingly scarcer with increasing carbon number. Although C-5+ hydrocarbons have been detected in the product mixtures of this reaction, high yields of such products were not obtained. An unwanted by-product of the process is pure carbon or coke, which can adversely affect the energy efficiency. In cases where CH_4_ needs to be valorised because of its remoteness and expensive/inefficient transport, even production of C_2_H_6_ C_2_H_4_ and C_2_H_2_ can be considered a sort of valorisation, however, safety issues need to be addressed as mentioned beforehand, especially for the case of acetylene. Firstly, these gases are much more easily compressible, making their transportation cheaper. Secondly, they can be transformed to liquids or other valuable products in a subsequent process. In some cases, even pure carbon products can be considered valuable in some forms, *e.g.* graphene nano-flakes^83^ and sheets.^84^ For a much more in-depth review of this particular process, the reader is referred to an extensive recent review by Scapinello *et al.*^85^

**Fig. 1:**
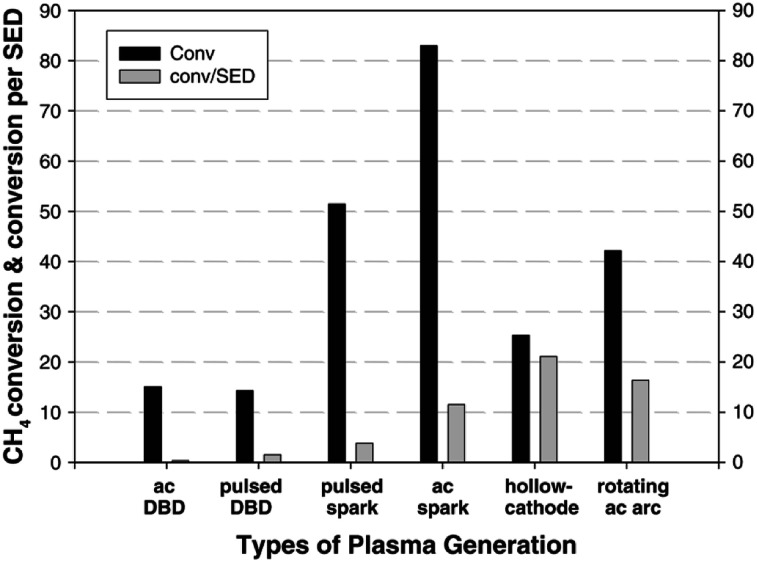
Comparison of CH_4_ conversion rate and associated energy efficiencies for various plasma reactor types. Reproduced from ref. 77 with permission from Springer Nature, copyright 2013.

The product selectivity of the process depends greatly on the gas temperature of the plasma, many times referred to as the “plasma warmness”. This can be seen in many studies discussed below, and is directly demonstrated by Lee *et al.* in a rather extensive experimental study.^77^ The authors showed a clear distinction between cold plasmas such as DBD and warmer plasmas such as spark, gliding arc, hollow cathode and rotating arc in terms of product distribution. Most of the product consists of either C_2_H_6_ or C_2_H_4_ and C_2_H_2_. C_2_H_6_ can be formed by the recombination of CH_3_ radical species, while C_2_H_2_ and C_2_H_4_ can be formed respectively from CH and CH_2_ intermediates, and also by dehydrogenation of C_2_H_6_.^77^ However, much higher electron energy is required for the dissociation of CH_4_ to CH and CH_2_. There are indeed some electrons present in the system with such high energies (>10 eV), but they are at the higher end of electron distribution and their concentration being very low, most of dissociation produces CH_3_. The main source of CH and CH_2_ species in the presented example is therefore assumed to be thermal dehydrogenation. Nevertheless, thermal gas phase dehydrogenation of the formed hydrocarbons is also possible resulting in consequent conversion of C_2_H_6_ to C_2_H_4_ and C_2_H_4_ to C_2_H_2_. Therefore, C_2_H_6_ is the main product in cold plasma systems due to a lack of further dehydrogenation mechanism. In warmer plasmas, C_2_H_2_ and C_2_H_4_ are more common. The study also demonstrates that warmer plasmas can convert CH_4_ into value added hydrocarbons at lower energy consumption. The efficiency of CH_4_ or CO_2_ activation in plasma is usually expressed in terms of specific energy input (SEI), which is stated as the ratio between the plasma power and the total gas flow rate.^86^ Conversion rate and energy efficiency for CH_4_ conversion in various atmospheric pressure plasma reactor types is compared in Fig. 1.

Non-oxidative CH_4_ coupling has been performed in multiple systems, gliding arc being one of them.^87,88^ In ref. 88, approximately 40% CH_4_ conversion was achieved with 20% selectivity towards C_2_H_2_ and 40% towards H_2_, which were the main products besides pure carbon and power was found to have little effect on the product distribution. When nitrogen was used alongside CH_4_, a maximum of 65% conversion of CH_4_ was achieved at 20% nitrogen with CH_4_. Regarding C_2_H_2_, maximum selectivity of 60% was achieved at a 50 : 50 N_2_ : CH_4_ ratio. The reason for higher conversion in the presence of added N_2_ was that the vibrational excitation of N_2_ to its meta stable states [(N_2_(A) and N_2_(a′))] will help with the activation of CH_4_. Even though N_2_ has relatively higher dissociation energy, the above mentioned meta stable species can increase the rate of CH_4_ dissociation to various CH_*x*_ radical species by transferring the potential energy to CH_4_ and thereby reduce the energy consumption. In another study, at a rather high specific energy input (SEI) of 2.09 MJ mol^−1^, 43% CH_4_ conversion was achieved with C-2 selectivity of 87% inside a gliding arc reactor.^87^ Influence of Ar addition on CH_4_ conversion was also studied, which determined that Ar addition into CH_4_ plasma reduced the energy input. It was revealed that a suitable percentage of Ar in the gas feed can increase the CH_4_ conversion by increasing the electron density in the reaction medium. However, both an increase,^88^ and decrease^87^ of C-2 hydrocarbon selectivity with Ar addition were reported. Nevertheless, no liquid products were detected in both cases.

Similar products (C_2_H_2_, H_2_ and C) were reported in studies related to non-oxidative CH_4_ coupling using a spark reactor.^77,89,90^ In a spark plasma channel with gas temperature in the range of 420–460 K was observed that lower reactor diameter and longer residence times increased the CH_4_ conversion but also the selectivity towards graphitic carbon deposition. Furthermore, the energy cost of C_2_H_2_ was lower at shorter residence time, which was about 12.1 kW h kg^−1^ under the optimized reaction conditions.^90^ The high selectivity towards C_2_H_2_ in plasma CH_4_ conversion is explained by a model considering more than 50 reactions and thermal balance and can be found elsewhere.^91^ It was observed that using gliding arc plasma could provide up to 35% improvement in conversion and energy efficiency when compared to pulsed plasma with parallel electrodes, most likely due to the distribution of the discharge over a larger gas volume.^92^

Pulsed microwave plasma (with gas temperature 1500–2500 K) was also used for C_2_H_2_ production, where the authors emphasized the importance of pulse length for conversion and selectivity.^93^ When pulse length was optimized at 60 microseconds at 1 kHz, CH_4_ conversion of 90% with 80% C_2_H_2_ selectivity was obtained. It was experimentally validated that by controlling the pulse duration, the densities of CH species and atomic H species in the system could be controlled which indeed influence the reaction pathways. Another study revealed that corona discharge can provide better C_2_H_2_ yield and higher CH_4_ conversion compared to that of DBD reactors at a given discharge power. This is ascribed to higher electron energy in the case of the corona reactor (10–20 eV) than in the case of the DBD reactor (1–10 eV).^94^

All of the aforementioned systems had somewhat thermal characteristics, and not much liquid products were detected. Experiments done in DBD were more promising in this regard, because a partial selectivity shift from C-2 to higher hydrocarbons can be observed when using colder plasmas. Yang *et al.* detected significant amount of propane and butane in DBD, and noticed a decrease in higher hydrocarbons formation as the SEI was increased.^94^ A screening of temperature and residence time on product distribution was presented in^95^ and it seemed that formation of C-5+ hydrocarbons peaked at the temperature of 200 °C. However, the major products were still C-2 hydrocarbons with a selectivity of over 70%. The authors also reported an enhanced conversion by 22% when CH_4_ was diluted with He, where CH_4_/He ratio equalled unity. This was attributed to an improved charge and energy transfer by added He into the system.

The effects of noble gas addition on CH_4_ conversion were further extended by Jo *et al.*^76^ Significant effect of diluent gases (He, Ne and Ar) on the conversion of CH_4_ was reported at a CH_4_ concentration of 10%. Noble gas addition did not affect the electron density, but it greatly affected the electron temperature, which explains higher CH_4_ conversion. Upon adding Ar, the conversion was the highest and almost double compared to that of He addition. In their next study,^96^ the effects of Kr and Xe were also compared and the study revealed that these gases had a significant effect on CH_4_ conversion. Kr addition resulted in the highest CH_4_ conversion, followed by Xe. On the other hand, He addition exhibited the highest selectivity towards C-3+ hydrocarbons, but the conversion was low. The difference in the selectivity and conversion depending on the noble gases in used in the system could be ascribed to their difference in the ionization cross section. Significant selectivity towards C-4+ hydrocarbons (∼10%) and propane (10–15%) were reported by Indarto *et al.*^97^ In this study, the C-2 selectivity was between 25 and 35%. It was noted that a high residence time favours the formation of shorter hydrocarbons (like C-2 or even pure carbon) and hydrogen. Another study dealt mainly with the effects of power, residence time of the gas in the reactor and discharge frequency.^75^ Both residence time as well as discharge power had lowered the energy efficiency as they were increased. Discharge frequency did not have much effect on either the energy efficiency or product selectivity. However, it affected the CH_4_ conversion and product yield, the highest being observed at 20 kHz. At this frequency, the maximum conversion of CH_4_ (25.2%) was observed at a CH_4_ flow rate of 50 mL min^−1^ and 45 W discharge power. In another study, residence time showed a big effect on C-2 and C-3 selectivity. C-3 selectivity increased with a longer residence time, while C-2 selectivity decreased, thus it is possible to control the product distribution merely by regulating residence time.^98^

Among many factors, discharge gap (the distance between the electrodes) is another crucial parameter that significantly influences the product formation in plasma assisted CH_4_ conversion processes. For instance, by changing the discharge gap, the rate of carbon deposition has been successfully controlled – it increased as the discharge gap increased.^98^ At the optimal discharge gap of 0.4 mm, combined selectivity towards C-2 and C-3 products of 80% with 25% CH_4_ conversion was reported at discharge power of 25 W. A study reports a modified DBD system with an electrode with disks attached at 5 mm intervals, effectively prolonging the discharge zone but decreasing the plasma density.^99^ The authors found out that with the same power but higher plasma density, the product selectivity was mainly in favour of C_2_H_6_ in a normal DBD, while the configuration with disks resulted in more C-3+ hydrocarbons.

A relatively simple kinetic model consisting of 8 species was developed,^100^ and was successfully fitted to the experimental data for non-oxidative CH_4_ coupling. A neural network model was also developed to determine the importance of different parameters on the product formation,^101^ by “teaching” the network using numerous data from multiple experimental studies. Using the model, it was found out that discharge power and gas flow were the most crucial parameters for the process, and that discharge frequency had next to no effect on the product distribution and conversion. While most other studies confirmed this predicted effects of flow and power,^94,98^ some studies reported a small but still significant effect of frequency.^88^ Note that this model was only fitted to experiments performed in non-oxidative CH_4_ coupling, while for example in oxidative coupling, it was shown that frequency can play and extremely important role.^102^ Models such as this one in the reference could be a very important part in further experiment designs. It has to be noted, however, that there were still many discrepancies between various models and experimental studies.

### 4.2 Partial oxidation of CH_4_

The partial oxidation of CH_4_ in plasma seems like a promising way of forming oxygenates. The reaction is initiated by electron collision with the neutral CH_4_/O_2_ molecules, forming both active oxygen derived and CH_4_ derived species. Major dissociation intermediates in this process are CH_3_ radical and O atom species. Articles discussing CH_4_ oxidation modelling^103–105^ go into greater depths of the mechanisms. It is of great importance to consider avoiding total combustion routes which form water and CO_2_, wasting both reagents. In gliding arc, for example,^70^ almost 100% selectivity towards syngas was achieved at O_2_/CH_4_ ratio of 0.6. While syngas can also be converted to liquids in subsequent processes, the focus of this review is more on the direct approaches. Therefore, thermal plasma characteristics should be avoided for this process and it should come to no surprise that most works published on the topic made use of DBD plasma.

**Fig. 2:**
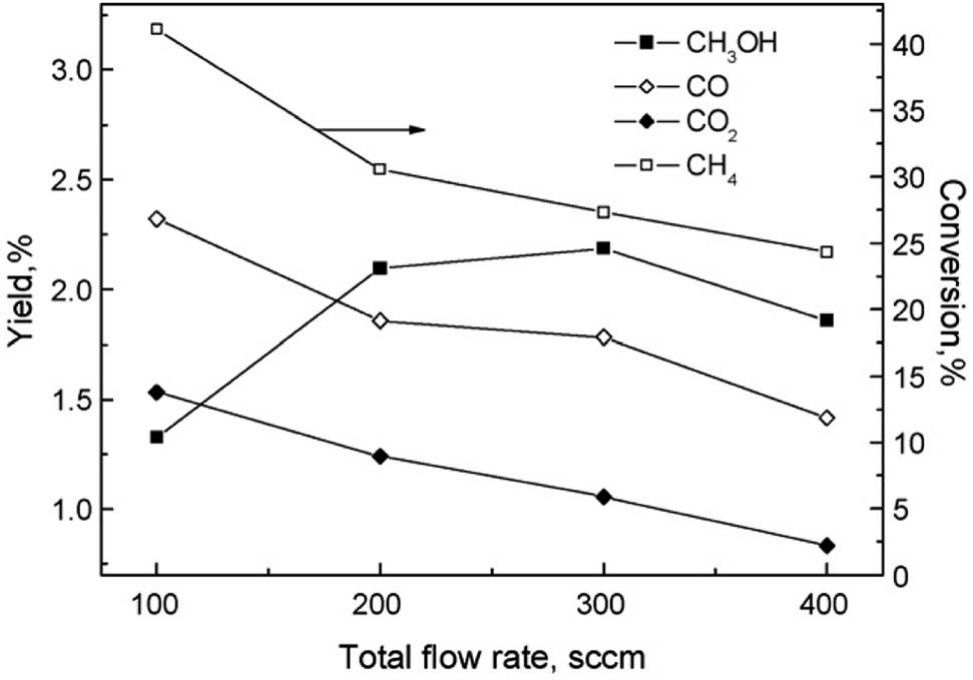
The effect of flow rate on product distribution (140 W, 7 kHz, 0.5 mm gap distance, ambient temperature, 1 bar, 1 : 1 CH_4_ : O_2_). Reproduced from ref. 107 with permission from Elsevier, copyright 2009.

Another factor to consider is the decomposition of CH_3_OH in plasma, and the shifting selectivity towards total combustion products as the residence time increases, as described in detail in a review by Indarto.^106^ It is noted that the optimal residence time should be found so that the yield of CH_3_OH or other oxygenates is maximized, as there is a compromise between CH_4_ conversion and organic oxygenate selectivity in regards to the specific energy input (SEI).^107^ The SEI can be adjusted by controlling the total gas flow rate at the same power, as illustrated in Fig. 2.

For this process, the ratio of CH_4_ and oxygen is extremely important. It was proposed in^108^ that an oxygen concentration of 15% is optimal, because formation of CO and CO_2_ was favoured as the oxygen content increased further. At this ratio, maximum CH_3_OH yield of 3% was reported, with its selectivity being 30%. Using air instead of oxygen, a reduced yield of 2% was obtained. C_2_H_6_, C_2_H_4_, propane and ethanol were also detected in the products mixture, although at lower concentrations. The addition of inert gases, namely He and Ar, to the CH_4_/O_2_ mixture was also studied.^109^ A 2.5 times increase in CH_3_OH yield at 2.5 dilution ratio was observed. With further dilution, CH_3_OH selectivity decreased and C_2_H_6_ selectivity increased. As the Ar content in the gas feed increased, higher electron density was measured, which was responsible for the improved conversion rate and product selectivity. The effects of noble gases on the plasma properties and associated plasma chemistry were already discussed along with non-oxidative coupling reaction. Aghamir *et al.* found that at their operating conditions, C_2_H_4_ was the major product when the voltage is below 12 kV.^110,111^ As the voltage was increased to 18 kV, the selectivity to CH_3_OH could rise to up to 60%. At voltages higher than 14 kV, there was three times as much CH_3_OH in the product compared to C_2_H_4_. It should be noted that the increase in applied voltage does not induce any significant change in the internal electric field due to the formation of space charge in the region between the electrodes. Instead, it can increase the number of available electrons in the system and influence the gas phase reactions.

Hydrogen peroxide is also considered as a wanted product besides liquid organic oxygenates.^112^ A yield of 29.2% H_2_O_2_ was reported for CH_4_ partial oxidation in plasma. The yield was similar as in the case of H_2_/O_2_ plasma reaction, but the SEI was ∼10 times higher in the case of CH_4_. Also, up to 57.3% selectivity towards liquid oxygenates was reported, which mainly consisted of CH_3_OH, formaldehyde and formic acid. A dependence of the selectivity on SEI was also made in another study using a CH_4_/O_2_ mixture.^113^ It was found that there is a maximum to CH_3_OH and HCHO selectivities at 8 kJ mol^−1^, with 64% combined selectivity at this rather low SEI. In the same study, the reaction of CH_4_ with iodine in plasma for the synthesis of methyl iodide was reported, the selectivity being about 95%. Methyl iodide is a useful intermediate for further processing as it can be easily hydrolysed into methanol in alkaline media. As an alternative to O_2_, air, halogens and nitrous oxide were also used as efficient oxidizing agents.^114^ With an inlet of 90% Ar, 5% CH_4_ and 5% N_2_O, a total 10% yield of CH_3_OH and HCHO was measured with 40% combined selectivity. However, it is more convenient to use oxygen or air as the oxidizing agent in terms of commercial point of view.

In the research by Larkin *et al.*, the previously addressed problem associated with the decomposition of liquid organic oxygenates within the plasma discharge zone was partially solved by using either *in situ* condensation by cooling the reactor^115^ or by using shorter residence time with condensation and recycle.^116^ In both cases, an increase in the yield of liquids, such as CH_3_OH, methyl formate, formic acid, formaldehyde and ethanol, was observed along with a total selectivity towards organic liquid products ranging from 24 to 52% depending on the reaction temperature. A similar approach can be found in the works of Nozaki, Okazaki and their colleagues.^57,117,118^ The authors used a cooled reactor (≤10 °C) to achieve *in situ* condensation of liquid products such as CH_3_OH, DME and formaldehyde. As these products condensed and were removed faster from the active plasma zone, there was a lower possibility of further decomposition. Therein, 70% and 30% combined selectivities of liquid organic oxygenates were observed when the yields were 5% and 20%, respectively, with the selectivity to syngas being about 40%. The authors remarked that a possibility of 30% one-pass yield with 80% selectivity might be possible if a syngas-to-DME reaction stage is added downstream. A kinetic modelling study of CH_4_ plasma oxidation was also performed to confirm the improved selectivity at lower temperatures.^119^ Simulations on CH_4_ partial oxidation in plasma, which were consistent with several experimental works, can be found elsewhere.^104,120^ This agreement seems promising for a better understanding of the process and future experiments in order to ensure a maximum selectivity and yield at minimum energy consumption.

### 4.3 Valorisation of CH_4_ by using water

Water being an abundant reagent, a process using it seems like a promising CH_4_ valorisation route. CH_3_OH synthesis from CH_4_ and water at lower pressures (133–133.3 × 10^2^ Pa) in a capacitively coupled plasma reactor was performed.^121^ Formation of C_2_H_6_, C_2_H_4_, C_2_H_2_, and CH_3_OH was observed along with carbon monoxide as the major product. Among the carbon containing species, CH_3_OH selectivity was heavily dependent on the reagent mixture ratio. A maximum selectivity of 20% was observed at the CH_4_ : H_2_O ratio of 1 : 5.

However, following studies mainly focused on the conversion at atmospheric pressure due to an ease of operation and scaling up. As an example, the process was performed in DBD^122^ where a CH_3_OH yield of about 1% was reported. By adding Kr (37.5%) to the reagent mixture, it was possible to enhance the conversions and CH_3_OH yield. Using a CH_4_ : H_2_O : Kr ratio of 3 : 1:2.4, the conversion was increased by ∼30% and selectivities increased from ∼30 to almost double for CH_3_OH, C_2_H_6_ and butane. The authors attributed these effects due to the possibility that Kr and Ar have very high energies of their lowest metastable levels (10 and 11.5 eV), so they can cause the dissociation of CH_4_ and H_2_O by energy transfer to those molecules. Kr and Ar also have deep valleys in momentum-transfer cross section at the electron energies of 0.5 and 0.2 eV, respectively, which can vibrationally excite CH_4_ and H_2_O. No such effects were observed with the addition of He, which is missing a valley in its momentum-transfer cross-section profile.^123^ As an alternative explanation, it is well established that the addition of noble gases into the plasma can significantly increase the electron density in the system which in turn enhances the plasma ionization and dissociation rates.^124,125^

### 4.4 Plasma coupling of CH_4_ and CO_2_

While most research on simultaneous conversion of CH_4_ and CO_2_ was done on the process of dry reforming,^126–129^ some works focusing on liquid chemicals were also carried out. While dry reforming was mainly achieved in warmer plasmas, such as spark or gliding arc, the research aiming for liquids was mainly performed in DBD. In one of such studies,^130^ CH_3_OH and ethanol, along with various carboxylic acids such as formic, acetic, propanoic and butanoic acids were detected in the liquid products. Among the gaseous products formed, syngas was prominent (89%) along with lower amounts of C-2 (8.5%) and C-3 hydrocarbons (1.5%). The authors proposed that plasma dissociation of a CH_4_–CO_2_ mixture mainly produces CH_3_, H, CO and O species, which were converted into products. Additionally, possible mechanisms were proposed by means of DFT calculations. For example, it's possible for acetic acid to be produced by either subsequent additions of CO and OH to methyl radical, or an addition of COOH. It is speculated that COOH is mainly formed by recombination of CO and OH radicals.

The process was also performed using stationary gas phase and high SEI (1400 kJ mol^−1^) in ref. 131. The authors observed the formation of long branched hydrocarbons, as at the longer reaction time at tested conditions, polymerization analogue took place. This could be explained as the addition of CH_*x*_ species on to the long chain hydrocarbon molecules within the reaction media. During the experiments, approximately 20% of the products obtained were non-volatile liquids whereas 1% of the liquid fraction also consisted of oxygenates, such as alcohols, ketones, esters and acrylic acids. In another study,^132^ methane dry reforming was integrated with a coal pyrolysis process. The authors noted a significant increase in yield of tar (13.3%), which was ∼1.5 higher than the yield when no plasma was used.

The selectivities between oxygenates can be shifted by varying the discharge gap inside the DBD reactor, as observed in the case of non-oxidative coupling. When a gap of 1.1 mm was used, the conversion of CH_4_ and CO_2_ was higher, and the formation of acids and liquid hydrocarbons was favoured. When this gap was increased to 1.8 mm higher amounts of ethanol and CH_3_OH were formed.^133^ It is evident from these and other reported results that the discharge gap can influence the selectivities, however, the exact changes in the conditions are not well elucidated. For example, it is possible that the power density increases when the electrodes are at a shorter distance. In another work,^128^ a 5.2% selectivity towards acetic acid was observed at CH_4_ : CO_2_ ratio of 2 : 1, with 64% and 43% of their respective conversions. Low amounts of ethanol (∼1.8%) and propanoic acid (∼1%) were also measured. In general, the overall selectivities towards liquids were low in the presented examples, and the major product in most cases was syngas. It is possible to obtain large amounts of CO in the gaseous product mixture as a result of CO_2_ dissociation, which would then be released as a major product.

### 4.5 Other CO_2_ conversion processes

Apart from CH_4_, hydrogen was used for CO_2_ reduction in several studies.^134,135^ A 85% CO_2_ conversion with 6% energy efficiency was reported at a H_2_ : CO_2_ feeding ratio of 3 : 1.^135^ The study focused mainly on the reverse water-gas shift process, and thus the product was syngas. Although the production of syngas is not negligible in the gas to liquid agenda, as discussed below, a more direct conversion was investigated.^134^ A surface discharge reactor was used to convert a mixture of 50% CO_2_ in H_2_. Among the products, CO, DME and CH_4_ were formed in approximately equal concentrations. Reactions of CO_2_ with water inside various plasma reactors were also studied in detail.^136,137^

Oxalic acid and H_2_O_2_ were detected from a mixture of CO_2_ and H_2_O in microwave plasma reactor.^138^ In another study by the same group, CH_3_OH was also formed – both during the reaction of H_2_O and CO_2_ and also during water plasma cleaning of the deposited organic molecules in the reactor.^137^ The authors proposed that pressure is a very important parameter which affects CH_3_OH selectivity. It was observed that the CH_3_OH yield increased by a factor of about 3.5 when pressure was raised from 240 to 400 Pa. A negative corona discharge was used in water and CO_2_ to produce ethanol and CH_3_OH in approximately 3 : 1 ratio.^136^ At the pressure of 1 atm and the temperature of 105 °C, their combined yield reached 4.7%. As the pressure was increased to 4 atm, a yield of 11.9% was observed. The authors speculated that the above mentioned alcohols formed from anionic species generated by the electron attachment to water and CO_2_ (H_2_O^−^ and CO_2_^−^ respectively).

Overall, it is evident that non-oxidative CH_4_ conversion process yields mainly C-2 and C-3 hydrocarbons. It also has the disadvantage of coke production. In terms of useful liquid chemicals, it seems that this process might be most useful in a two stage systems where the resulting C_2_H_2_ or C_2_H_6_ could be converted downstream into liquid chemicals. Regarding direct conversion to liquids, using different oxidants in order to get organic oxygenates such as alcohols, aldehydes and carboxylic acids seems to be more promising. Using CO_2_ as the oxidant seems to produce mainly syngas and is not the most effective in this agenda. It should be noted that syngas can indeed be converted to liquids in a downstream process, but this presents additional investment and operational costs, and thus direct conversion methods are more welcome. Naturally, there are additional costs associated with direct methods as well, namely the need to perform recycling and other drawbacks when not being able to achieve high single-pass yields. Using water with both CO_2_ and CH_4_ seems to produce significant yields of oxygenates. However, more research needs to be done in this field. The most promising process seems to be the partial oxidation of CH_4_ using oxygen, where high yields of oxygenates were reported. It should be noted that many discrepancies between different experimental works have not yet been fully elucidated, so a better understanding of plasma mechanisms and pathways is also needed to optimize the experimental work. All the above processes can be performed in coupled plasma-catalytic systems as well, which may further increase the yields and energy efficiencies. Works dealing with plasma-catalytic conversion of either CO_2_ or CH_4_ are discussed further in the review.

## 5. Plasma-surface interactions

A material placed in plasma experiences a large number of complex interactions with various excited species generated therein. Such physical and chemical interactions between plasma and the catalyst embedded in the reactor can significantly affect the yield and selectivity of product formation. Thus, prior to the effects of a catalyst in plasma, it is worth mentioning the possible elementary plasma–surface interactions from the perspective of plasma catalysis.

### 5.1 Adsorption

Adsorption is one of the most important surface processes in heterogeneous catalysis. The adsorption of plasma reactive species onto the surface of the interacting materials generally occurs either *via* physical adsorption (physisorption) or *via* chemical adsorption (chemisorption). Physisorption is facilitated by weak van der Waal's forces between the impinging particle and the interacting surface. Physisorption is a very weak and exothermic (Δ*H* = 1–25 kJ mol^−1^) interaction, which weakly binds the molecule on the surface.^139^ Even though the associated interactions are very weak, physisorption can sufficiently increase the lifetime of the incoming species in the proximity of the catalyst surface. This can significantly improve the performance level in plasma assisted conversions.

Contrary to physisorption, the energy associated with chemisorption is much higher (Δ*H* = 40–400 kJ mol^−1^). When neutral plasma species reach the vicinity of the surface, electronic interactions are possible. Theoretical calculations show that the chemical interactions between valence states of the surface atoms with the incoming species are the primary reason for the chemical adsorption on the surface. This observation justifies the difference in the catalytic activity and selectivity of various transition metals in catalytic or hybrid plasma catalytic systems.^140,141^ Furthermore, chemisorption of vibrationally excited molecular species on the catalyst bed is one of the major reasons for the increased reaction kinetics in hybrid plasma catalyst systems.^80^ Such phenomena are experimentally validated through optical emission spectroscopic analysis.^142^

In addition to neutral atoms and excited molecules, plasma contains charged species including ions and electrons. Due to the lower mass, the mobility of the electrons in the system is much higher compared to that of positive ions. Thus a material floating in plasma acquires a negative potential. Report by Li *et al.* reveals that the negative charge accumulated on the electrode surface during the discharge remains for several minutes even after the plasma is turned off.^143^ This is an after effect of trapping of adsorbed electrons on the material surface. These trapped electrons are able to act as an electron reservoir and generate surface streamers. Such surface streamers can significantly improve the conversion rate in plasma catalysis, which is discussed in the following sections. On the other hand, due to lower energies, the influence of ions is usually disregarded in reactors operated at atmospheric pressure.

### 5.2 Recombination

Plasma gains its potential energy by means of inelastic collisions, leading to various processes including excitation, ionization or dissociation. This excess energy will be released back into the system in the form of radiation by the successful recombination of various energetic species. For instance, the energy released during the recombination of 2 atomic oxygen to form molecular oxygen is estimated to be around 5 eV.^144^ Recombination of various species in the gaseous phase is well presented in literature and is of greater importance in catalyst free activation of CH_4_ and CO_2_.^113,139,145,146^ However, the recombination on the surface of a solid material is largely influenced by its surface recombination coefficient (*γ*). Surface recombination coefficient for a solid material depends on its chemical composition, impinging plasma species and the surface temperature.^147,148^

Three different mechanisms are commonly used to explain such reactions on the surface namely, Langmuir–Hinshelwood (LH) mechanism, Eley–Rideal (ER) mechanism and Mars–van Krevelen (MvK) mechanism. In the case of LH mechanism, the reaction occurs between two species which are in adsorbed state. Contrary to this, in ER mechanism a preadsorbed species reacts with an impinging species in the gaseous phase. LH mechanism prevails at medium temperature. Low surface diffusion coefficients and the high surface site coverage restrict LH mechanism at lower temperature.^149^ At elevated temperature, a shift from LH to ER mechanism can be observed due to higher desorption rate.^150^ The MvK mechanism is described by the incorporation of constituents from the catalyst lattice into the reaction by-products. However, due to the lack of consistency and accuracy of this mechanism, attention has been given predominantly to LH and ER mechanisms in heterogeneous catalysis.^151,152^

### 5.3 Ion implantation and sputtering

Ion implantation is the process in which high energy ions in the plasma penetrate into the bulk of a material. Such phenomena can alter the physical, chemical or morphological characteristics of the catalyst embedded in the discharge chamber. However, this process requires very high ion kinetic energy (a few keV).^153,154^ Sputtering is strikingly different from ion implantation processes, where the surface atoms or molecular species are knocked out by high kinetic energy of the incident ions. For sputtering to happen from a solid surface, the incident ion should have a kinetic energy above the binding energy of the surface atoms. However, both ion implantation and sputtering are very unlikely to happen in generally used atmospheric pressure plasmas for CH_4_ and CO_2_ conversions.^151^

### 5.4 Deposition and etching

Plasma enhanced deposition techniques are well known for large scale production of various carbonaceous structures.^155–157^ However, carbon deposition during plasma assisted conversion of CH_4_ is a serious issue that decreases the energy efficiency and catalyst deactivation. The problems associated carbon deposition during both catalytic and plasma assisted catalytic conversion of CH_4_ is addressed elsewhere.^158–160^ It is considered that the dissociation of CH_4_ over the catalyst surface yields active monoatomic carbon.^161^ This carbon is generally removed into CO or CO_2_ by reacting with oxygen in the system. If the rate of carbon removal is less than the rate of its formation, carbon will polymerize on the catalyst surface. This process of carbon deposition, so-called coking, can adversely affect the activity and efficiency of the catalyst.

One of the possible methods to reduce coke formation is to add small amounts of sulphur on the catalyst surface. However, the supply of H_2_S onto the catalyst surface can permanently deactivate the catalyst. Another approach is to alloy the catalyst surface with tetra- or penta-valent p metals such as Ge, Sn, Pb, As, Sb or Bi to avoid the metal-carbide formation on the surface, which is the precursor for the coke formation.^162^ Nevertheless, alloying can adversely affect the activity of the catalyst and reduce the conversion rate and selectivity. The most acceptable way to avoid coking is to use metals which are resistant towards metal–carbide bond formation.^162–164^

Contrary to deposition, etching is a process by which solid atoms or molecules are converted into small volatile molecules by reactive plasma species.^165^ Plasma etching can be used for the removal of carbon deposited on the catalyst surface, in order to recover the catalytic activity. This can be achieved *in situ* by leaking sufficient amount of O_2_ into the system. Nevertheless, etching of active metals or inorganic supports used in plasma conversion does not happen as these materials form only solid by-products on reacting with the plasma reactive species.

The major challenge of the plasma research field is to elucidate the specific roles of each type of interaction and distinguish them on the basis of numerical values. For example, the prediction of various constants such as adsorption and recombination coefficients, ionization and dissociation constants in the presence of a catalyst within the discharge zone, and how the physical and chemical properties of the catalysts influence these values. Since plasma is a complex mixture of various species and the catalyst used for a specific reaction can be very unique in terms of substrate, specific surface area, pore size, type of metal, size of metal particles, *etc.*, this remains a great challenge faced by the plasma community.

## 6. Plasma pretreatment of the catalyst

Plasma technology has been established as a key tool for the surface modification of various catalytic materials. Plasma treatment has proven to be a fast and efficient technique to replace calcination process, which is one of the most important steps in catalyst synthesis. Indeed, plasma treated catalysts exhibit smaller particle size and lower extension of undesired surface contaminates.^166^ Alongside, plasma assisted doping can be an additional benefit. Thus plasma processing is largely used for the pretreatment of various catalysts to induce physical or chemical modifications to improve their activity. Through various physical and chemical interactions, plasma can modify the surface morphology and chemical structure of the interacting material.

The physical modification is mainly associated with the changes in specific surface area or surface morphology, which in turn modifies the electronic properties of the material.^167^ The increase in specific surface area can originate either from improved dispersion of the active catalyst on the support or by creating wrinkled structures on the surface.^168,169^ However, plasma treatments have different effects on specific surface area for different catalysts.

**Fig. 3:**
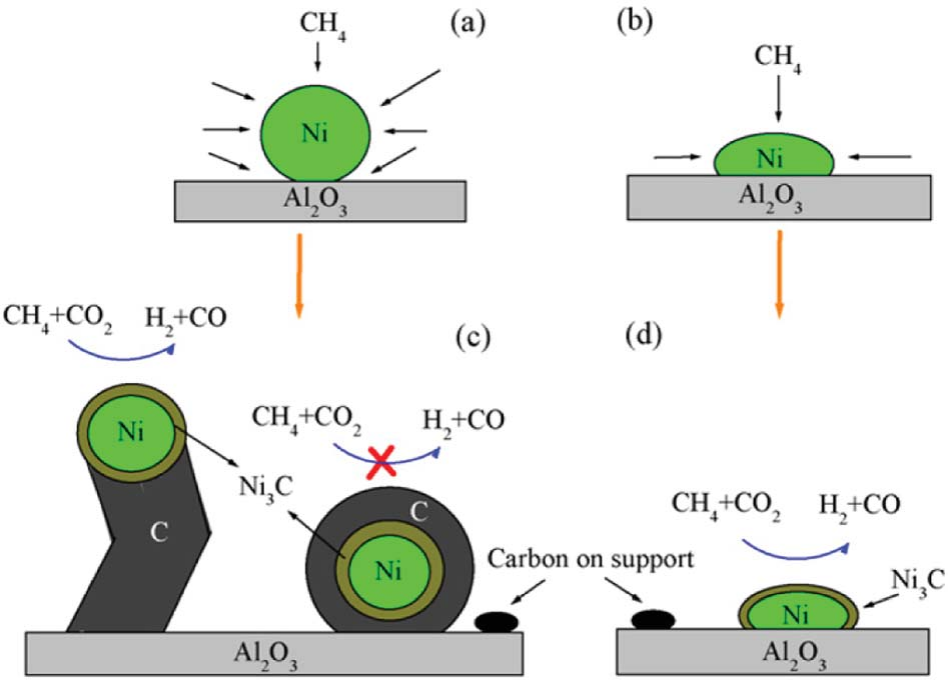
Schematic representation of CO_2_/CH_4_ reforming over (a and c) NiAl–C (calcinated); and (b and d) NiAl-PC (plasma treated prior to calcination) samples. Reproduced from ref. 161 with permission from Elsevier, copyright 2008.

One of the major applications of plasma physical modification of catalyst materials is to reduce coke formation and subsequent deactivation of the catalysts during the dry reforming reaction, which is speculated to occur due to the flattening of the catalyst after plasma exposure (Fig. 3).^161^ This is a cost-effective strategy to avoid the use of expensive noble metals for the prevention of coke formation. Other beneficial examples of plasma physical modification of the catalyst surface are provided in literature for various other processes as well.^170–176^

The underlying mechanisms on plasma induced morphological changes on various catalytic materials are studied in detail. It is assumed that trapping of electrons from plasma on the metallic particles creates a thin plasma sheath around it.^66^ Due to this, these particles will experience strong electrostatic repulsive forces from the flow of electrons from the surrounding plasma. This coulombic force of repulsion can cause elongation or distortion of the metallic particles on the surface.^177^ Alongside, plasma electrons can persuade faster nucleation and slower crystal growth of the metallic particles which in turn reduces the particle size.

As a chemical modification approach, plasma treatment is used as an appropriate technique for various oxidation and reduction reactions. In the case of catalyst design and fabrication, H_2_ plasma reduction of P_2_O_5_ in the presence of metal oxide precursors has been found to be efficient for the synthesis of metal phosphides used for hydrodesulphurization catalysis.^178^

In the context of CH_4_ reforming, reduction of NiO to active metal is well known.^179,180^ In a recent report, Rafik *et al.* presented a low temperature H_2_ plasma pretreatment of a Ce–Zr promoted Ni catalyst with the intention of improving its activity towards CO_2_ methanation.^181^ H_2_ plasma pretreatment can successfully replace the conventional thermal pretreatment and allows the reduction reaction to take place at ambient temperatures with lower hydrogen consumption.^181^ In another example, Ar plasma pretreatment of Ir/Al_2_O_3_ catalyst was used for the reduction of Ir ions into metallic Ir, which improved the catalytic activity towards CO_2_ reforming over CH_4_.^182^ However, plasma pretreatments may not necessarily induce the same chemical effects to different metallic species. For example, after a Co–MnO_*x*_ catalyst was exposed to DBD discharge, the relative ratio of M^4+^/Mn^3+^ and Co^2+^/Co^3+^ showed a notable increase.^183^ Thus induced chemical changes increased the electron transfer efficiency between Mn and Co species and thereby improved the NO catalytic oxidation activity.

In addition to the changes in oxidation states, plasma treatment can increase the concentration of chemisorbed O_2_, which can improve the activity of the catalyst. Further, heteroatom incorporation can be employed in order to improve the catalyst activity for applications such as oxidation, reduction, molecular abatement and reactions in fuel cells.^184–186^ Chemical reactions between the plasma reactive species and the catalyst material create surface defects and the incorporation of heteroatoms (doping), which can in turn effectively modify the electronic band structures and surface states of the material.^167,187^ Numerous other examples of chemical catalyst modifications using plasma can be found in literature.^188–193^

All the above mentioned processes are extremely important to research, as they take place during plasma catalytic processes for chemical synthesis. A good understanding of these mechanisms is extremely important to enable good experimental performance.

## 7. Synergistic effects in plasma assisted catalytic conversion

Possible interactions between plasma and a material placed inside the discharge zone have been discussed already. Indeed, when a catalyst is introduced into the plasma chamber, it can slightly or significantly change the properties of plasma. Nevertheless, the influence of a catalyst inside a hybrid plasma catalytic chamber originates from the interplay between numerous complex surface reactions. The synergistic effects inside the hybrid plasma catalytic system can be defined as the ratio of the degree of conversion in the presence of catalyst along with the plasma to the sum of conversions with the catalyst and plasma measured separately.^194^ This synergy can evidently influence the reaction kinetics, product yield and selectivity by providing an alternative reaction pathway. It should be noted that the presence of a catalyst does not necessarily induce a positive impact on the product yield or selectivity. Related examples are discussed later in this section.

**Fig. 4:**
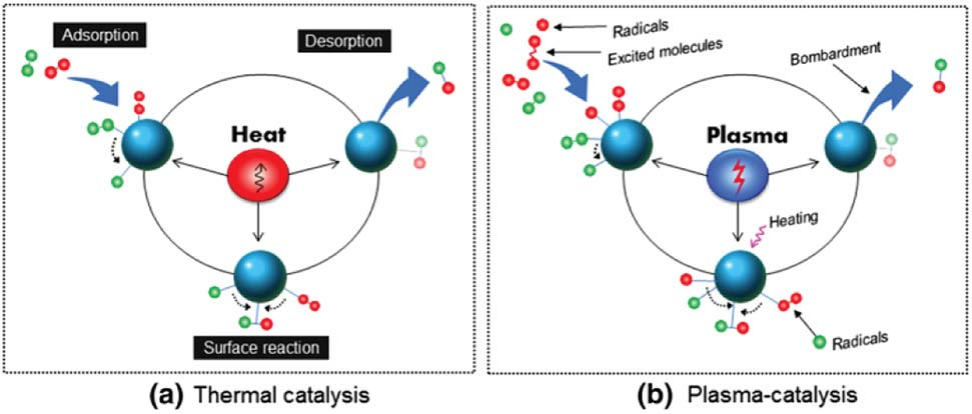
Schematic comparison of thermal and plasma catalytic surface processes. Reproduced from ref. 195 with permission from Springer Nature, copyright 2015.

In general, the role of a catalyst in thermal catalysis is to provide new reaction pathways, which might successfully reduce the energy of activation for the reaction. However, the effects a catalyst has on plasma are somewhat different compared to those it has on a thermal catalytic reaction. In general, catalyst is active at lower temperatures in hybrid plasma systems. Plasma is often used to achieve catalytic reaction even at room temperature. Dissociated atoms in plasma are highly electrophilic and easily get adsorbed at the electron rich centres on the catalyst surface, react among each other and desorb away.^195^ Also the chemisorption of plasma excited species is found to be faster and more efficient than ground state molecules. Another plausible reaction could be between the chemisorbed species on the catalyst surface and the dissociated atoms, radicals or excited species in the gaseous phase through previously described ER mechanism. In another sense, the surface reactions happening inside hybrid plasma catalyst chamber are more complex than conventional thermal catalysis and are still in a need of thorough investigation. A comparison of thermal and plasma catalytic process are schematically presented in Fig. 4.^195^

**Fig. 5:**
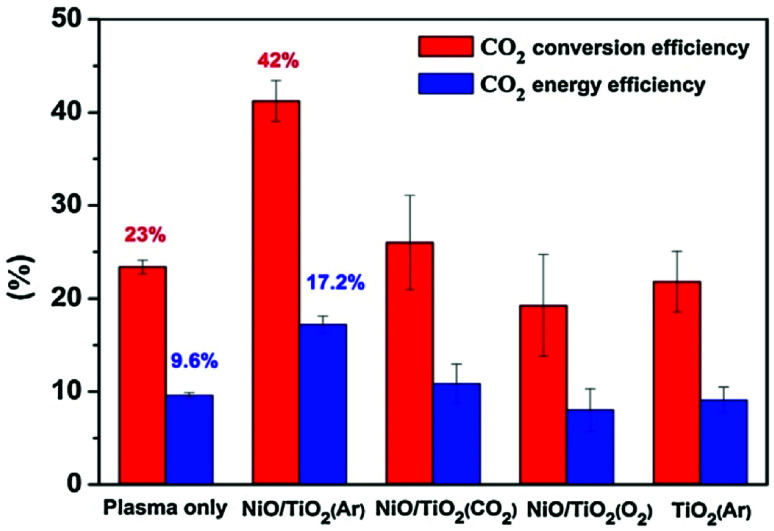
CO_2_ conversion and energy efficiencies, measured during the plasma-catalysis CO_2_ dissociation, are shown for the NiO/TiO_2_ catalysts prepared by plasma treatment with different gases (O_2_, Ar, CO_2_). Reproduced from ref. 198 with permission from Elsevier, copyright 2016.

The dissociation mechanism on the surface of an active material is explained differently in the presence and absence of plasma. When a CO_2_ molecule approaches the surface of NiO/TiO_2_ catalyst, it gets adsorbed at the oxygen vacancies and undergoes dissociative electron attachment (DEA).^196^ DEA is defined as the low energy electron induced formation of a negative metastable ion (in the present example: CO_2_^−^), which undergoes subsequent dissociation. On the other hand, when plasma is introduced along with the catalyst, the dissociation rate tends to increase.^197^ In one of the recent reports, the influence of various plasma pretreated NiO/TiO_2_ catalysts inside a microwave discharge for CO_2_ conversion clarifies the plasma-catalyst synergy.^198^ By introducing Ar plasma pretreated NiO/TiO_2_ inside the plasma chamber, the energy and conversion efficiencies were found to increase from 9.6% to 17.2% and 23% to 42% respectively. However, it should be noted that the efficiency of all the tested catalysts was not high enough to provide a positive impact as illustrated in Fig. 5. A significantly improved conversion under hybrid plasma catalytic system is assumed due to a higher threshold energy value for DEA cross section in gaseous phase (5–10 eV) compared to the one at the catalyst surface (1.7 eV).^198^ The effect could not be generalized for all the tested catalysts, as clear from the results presented in Fig. 5. This clarifies the complexity and lack of understanding in the grass root level in plasma catalysis.

Plasma-catalyst synergistic effects during CH_4_ activation were well studied in the past. Nozaki *et al.* explained the effects of plasma activation of CH_4_ in a DBD reactor with and without a catalyst packing.^80^ In a Ni/SiO_2_ catalyst only process, there was no CH_4_ conversion at temperatures below 400 °C and a spontaneous increase in the conversion rate was observed at temperatures around 600 °C. When the catalyst was coupled with plasma, strong synergistic effects yielded very high conversion rates (roughly >50% increase) in the temperature range between 400 and 600 °C.^80^

**Fig. 6:**
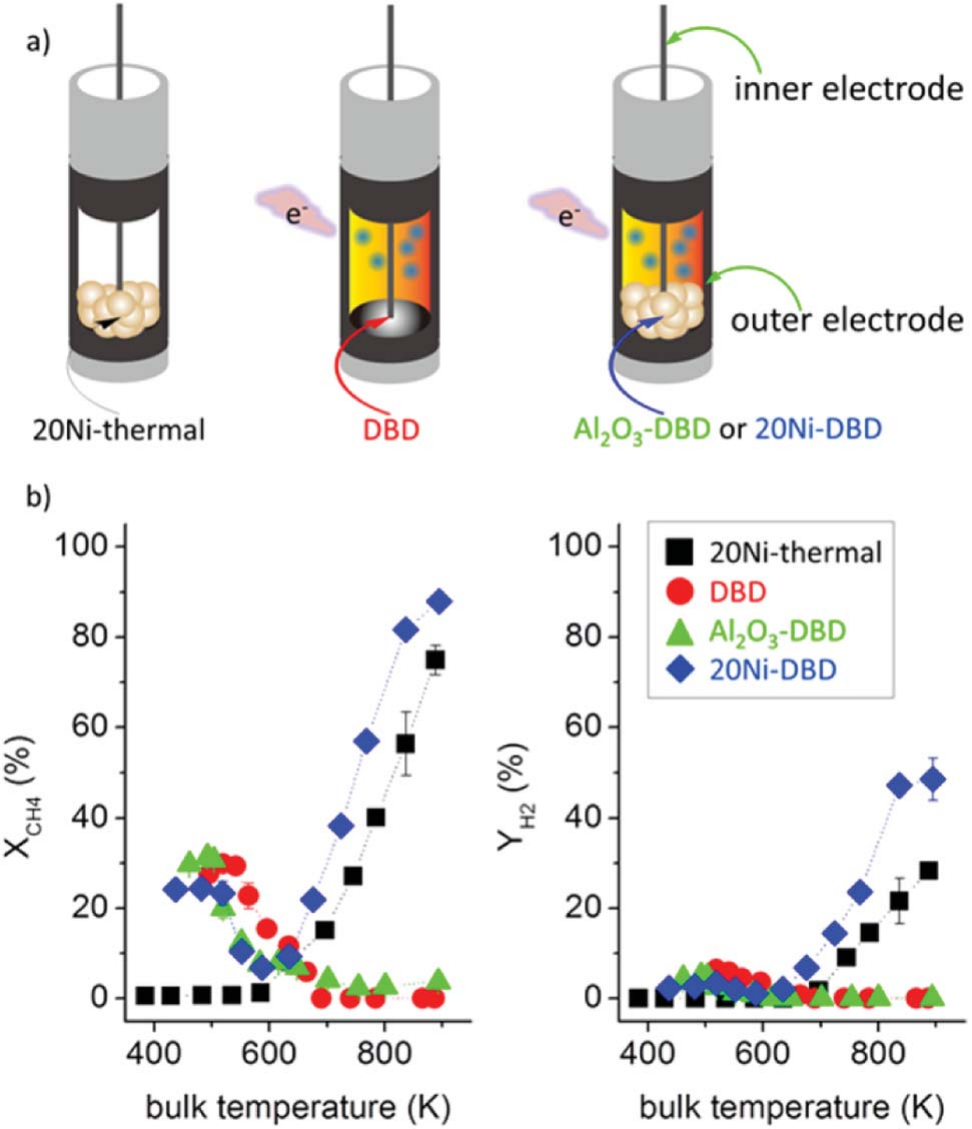
(a) Reaction environments for the CH_4_ reforming and (b) profiles of CH_4_ conversions (*X*_CH_4__) and H_2_ yields (*X*_H_2__) at various reaction environments obtained *via* bulk temperature controls. Reproduced from ref. 200 with permission from American Chemical Society, copyright 2016.

In direct plasma conversion, a very large amount of vibrationally excited CH_4_ existed in the system, which stayed non-reactive in the absence of any active catalyst. However, such vibrationally excited molecules can get adsorbed easily on the catalyst surface and get converted into products.^199^ In a very recent study, Kim *et al.* compared various aspects of CH_4_ conversion by considering: (1) gas phase dissociation of CH_4_, (2) dissociation in the presence of porous Al_2_O_3_ and Ni/Al_2_O_3_ catalysts, (3) thermal effects due to plasma and (4) interactions between Ni catalyst and excited plasma species.^60^ It is clear that the effect of temperature is predominantly seen in the presence of an active Ni catalyst. Temperature dependence on conversion in various tested conditions is compared in Fig. 6,^200^ which describes the plasma-catalyst synergy as a function of temperature. Additionally, higher surface area of the incorporated material further facilitates the vibrational excitation and subsequent degradation of CH_4_ by inducing higher bond polarization.^201^

Incorporation of the catalyst inside the plasma reactor does not necessarily increase the conversion rate or product selectivity in all the cases. For example, in a fully packed reactor, the formation of filamentary micro discharges will be significantly reduced due to the decrease in discharge volume.^202^ As a result, efficiency of molecular conversion can be reduced to a large extend. Another interesting example can be found in the report by Sentek *et al.*^203^ A catalyst free DBD reactor showed higher CH_4_ conversion compared to the one embedded with Pd/Al_2_O_3_ catalyst. However, it's worth mentioning that incorporation of the catalyst significantly increased the product selectivity towards C_2_H_6_. Similar examples of a reduction in molecular conversion and an increased product selectivity in catalyst packed plasma reactors can be found elsewhere.^202^ It is indeed clear that the effects of plasma on the catalyst are not invariably synergistic. The sum of individual effects of plasma and catalyst taken separately can be superior or inferior to the effect using plasma and the catalyst, which can be reflected in multiple outcomes including changes in conversion rate, energy efficiency or product selectivity.

## 8. Catalyst selection strategy for plasma-hybrid reactors

To date, there are hundreds of articles on plasma catalytic activation of CH_4_ and CO_2_. The most widely accepted strategy is to use a catalyst that is found efficient for conventional thermal catalysis. For example, Mo, Cu or Ni based catalysts can be used in thermal catalysis for the conversion of CH_4_ or CO_2_ into liquid fuels like CH_3_OH.^204,205^ Catalysts derived from the same group of elements are also efficient in hybrid plasma catalytic systems.^206,207^ Similar catalyst selection strategy has been applied for many other plasma assisted conversions including dry reforming, CO oxidation, CH_4_ conversion to syngas and molecular abatement.^202,208–210^ Nevertheless, no general rule has been established yet for the catalyst selection for plasma assisted conversion reactions. The activity of different catalysts inside the plasma chamber is determined by multiple factors ranging from chemical composition to physical properties. This section focuses on various effects that catalyst properties have on plasma, and how these effects can influence conversion rates, selectivities and energy efficiencies.

The position of the catalyst, embedded in the plasma discharge chamber, can largely influence the extent of various synergistic effects and thus the conversion rate. Based on the packing strategy, catalyst embedded plasma reactors can be classified into three classes, namely single stage, double stage and multistage plasma reactors.^211^ In a single stage reactor, the catalyst is partially or completely immersed in the discharge zone. In such a system, plasma particles directly interact with the catalyst and the catalyst can even change the discharge behaviour of the plasma.

In a double stage reactor, the catalyst bed is located downstream of the discharge zone. In many cases, single stage reactors were found better than double stage reactors for multiple purposes including dry reforming, volatile organic compound abatement and air purification.^211–213^ Certainly, this claim cannot be generalized. In contrast to single stage or double stage reactors, multistage plasma reactors are more interesting for industrial scale processing, which allows a stepwise activation or deactivation of various species.

In the case of atmospheric pressure plasmas, chemically reactive species have a very short lifetime, which is on the order of a few ns to several μs. For example, the estimated lifetime of an electron is as low as 10 ns whereas atomic O(^1^D) and OH radical species have lifetimes on the order of a few μs.^214,215^ O_3_ species having a much larger lifetime (a few minutes) can be attributed to their lower reactivity compared to that of atomic or radical species. Nevertheless, lifetimes of any species inside the plasma system are largely influenced by the medium surrounding the active species under examination.^215^ For an effective utilization of the reactive species in hybrid plasma catalyst systems, plasma should be as close as possible to the catalyst bed to enable direct plasma-catalyst interaction which allows efficient diffusion of the species onto the surface.^211^ The criteria for direct interaction between the plasma and catalyst can be expressed as,*Λ* = *l*/(*L*_D_ + *L*_ef_) ≤ 1where *Λ* is a dimensionless parameter. *l*, *L*_D_ and *L*_ef_ are the distance between the plasma and catalyst, diffusion length of the neutral atoms and migration length of the charged species under electric field respectively. From the equation, it can be elucidated that single stage plasma reactors enable better plasma-catalyst direct interactions compared to those of double stage reactors.

In plasma assisted conversion, the catalyst bed usually consists of an active material coated on a suitable support. Such supports can be mainly classified as semiconductors (metal oxides), ferroelectric materials or zeolites. The electrical properties of the catalyst are very important in determining the electrical properties of the plasma. For example, highly conductive materials are not the best candidates for hybrid plasma reactors. When a dielectric material is introduced into the discharge zone, charge accumulation on the surface of the catalyst creates a non-uniform distribution of the electric field.^216^ Such a distribution indeed enhances the electric field inside the plasma.

**Fig. 7:**
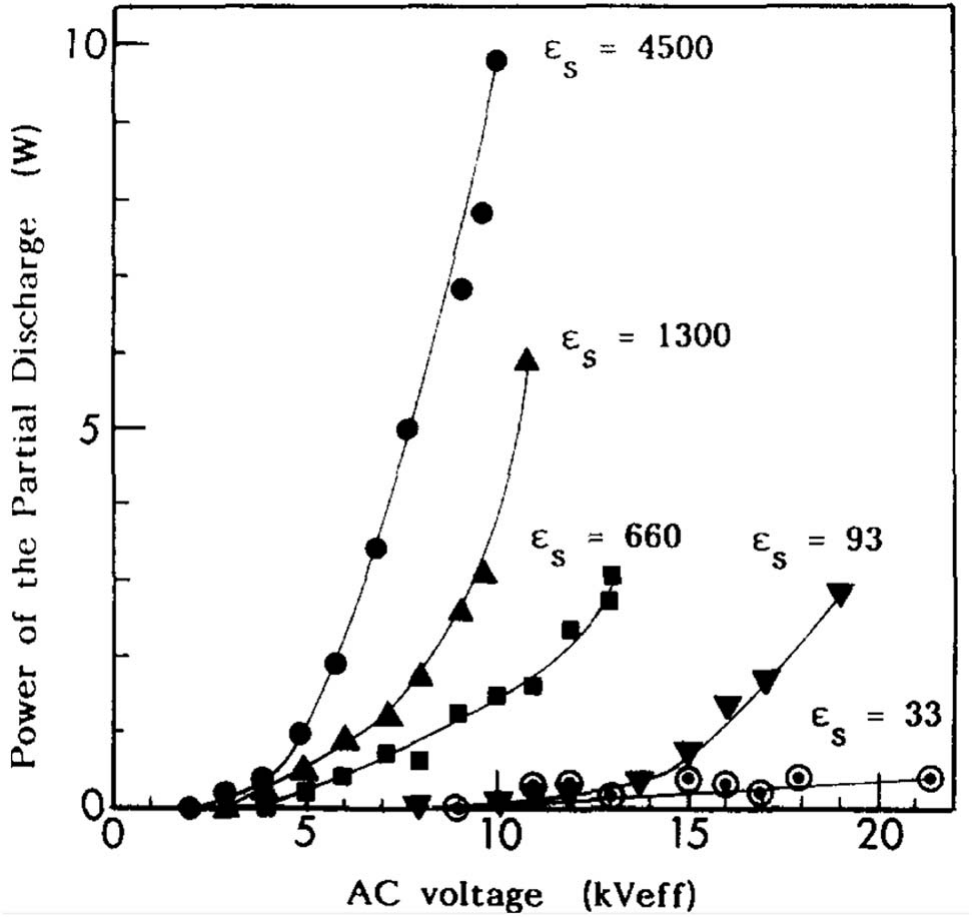
Power of the partial discharge in the packed bed with different specific dielectric constant *ε*_s_. Reproduced from ref. 219 with permission from Elsevier, copyright 1990.

When the catalyst is embedded in pellets, the effects are mainly influenced by the curvature, contact angle and dielectric constant. The enhancement of the electric field is much higher at sharp edges due to the electric edge effect.^217^ Furthermore, plasma parameters such as electron temperature and densities of radicals, ions and electrons show significant increase with the increase in dielectric constant of the packed material.^218^ As presented in Fig. 7, the increase in dielectric constant of the packing material and the applied voltage in an AC discharge favours an exponential increase in the power of the partial discharge.^219^ In some of the early studies, the molecular conversion rate in plasma was found to increase merely by incorporating a dielectric material in the discharge zone, which emphasizes the aforementioned influences.^220^

The shape and morphology of the embedded material is significantly important as it determines the properties of plasma and associated molecular conversion rate.^221^ Especially the influence of porous structures on the catalyst surface has to be addressed in this context. The formation of micro discharges inside the pores of a dielectric material placed in plasma is well known. Zhang *et al.* reported that inner regions of the pore are characterized by enhanced electric field strength, lower electron density and higher electron temperature, electron impact ionization rate and ion density and these factors can remarkably influence the plasma catalytic process.^222^ Nevertheless, pore size and discharge voltage were the key parameters that influenced the formation of micro discharges inside the pore.

**Fig. 8:**
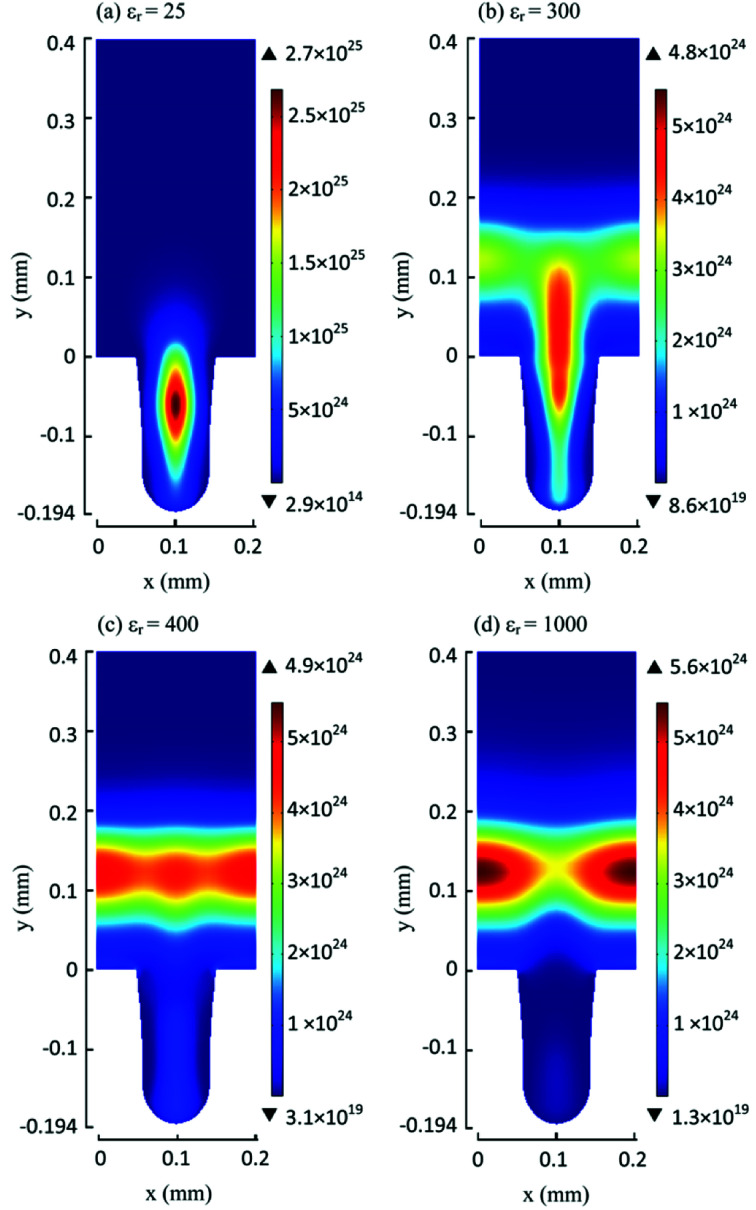
Distributions of the electron impact ionization rate, averaged over time in one AC cycle, for different dielectric constants: (a) *ε*_r_ = 25, (b) *ε*_r_ = 300, (c) *ε*_r_ = 400, and (d) *ε*_r_ = 1000, for a helium discharge sustained at 20 kV with a 100 μm pore. Reproduced from ref. 223 with permission from American Chemical Society, copyright 2016.

Theoretical investigations on the influence of the material dielectric constant and pore diameter on the plasma properties give better insight into the related issues.^223^ It is postulated that plasma generation inside large pores is enhanced in a broader range of dielectric constants whereas inside smaller pores the enhancement is mostly limited to lower dielectric constants. Thus the most commonly used catalyst supports such as Al_2_O_3_, SiO_2_ or zeolites (*ε*_r_ > 11) allow micro discharges inside smaller pores. On the other hand, ferroelectric materials such as BaTiO_3_ (*ε*_r_ ∼ 10 000) cannot yield plasma enhancement even inside pores up to a size of 100 μm.^223^ (Fig. 8) The paper gives an outlook into the formation of discharge inside the pores of a catalyst based on the pore size and material dielectric constant. Even though discharge inside the catalyst pores are still considered as one of the influential factor that determines the conversion rates and product selectivity, its role in plasma catalysis is not well defined yet. One major limitation of this paper is that the model is less applicable for many commonly used catalysts which are having nano porous structures.

The effects of metallic particles on plasma properties are studied extensively by many researchers. As reported by Jo *et al.*, the incorporation of Pt metal into Al_2_O_3_ increased the formation of CH_3_ radicals compared to CH_2_ or CH ones during CH_4_ discharge.^224^ This was attributed to the reduction in the electric field in the voids between the catalyst pellets along with the increased electric field in the proximity of Pt. This effect can be efficiently utilized in order to achieve better control over the type of reactive species in the plasma as well as the product selectivity.

Kim *et al.* studied the effects of metallic nanoparticles on surface discharge formation in the case of zeolites.^225^ The VOC oxidation ability inside an Ag/Cu-zeolite catalyst loaded plasma reactor tended to increase with metal loading. Firstly, metal loading can increase the plasma-catalyst interaction cross section. Additionally, metal particles on the surface enhance the electric field and allow the expansion of plasma over the catalyst surface, resulting in surface streamers. It is well known that physical and chemical characteristics of surface streamers on metal–zeolite catalysts are largely correlated with the type of metal and Si/Al ratio. Contrary to this, when the catalyst support is derived from BaTiO_3_, it yields micro discharges restrained to a lower volume.^226^ Even though surface streamers are assumed to be a necessity for a better molecular conversion in plasma catalysis, their physical and chemical characteristics have not been fully elucidated yet, although lots of strong efforts have been made recently. In particular, the coupled modelling and experimental study by Wang *et al.*^227^ inspected the formation of discharge in a packed bed reactor utilising different dielectric materials. The study uncovers three different types of surface discharges, namely positive restrikes, filamentary microdischarges and surface ionization waves. They noticed a clear distinction between the surface discharge type depending on the dielectric constant of the packing material. At low dielectric constants (*ε*_r_ = 5), there is a prevalence of surface discharge, which can be spread over multiple adjacent beads, while spatially limited filamentary discharges at bead contact points are preferred at high dielectric constants (*ε*_r_ = 1000). The authors emphasize the importance of such knowledge when designing plasma compatible catalysts and processes.

Thus in conclusion, the physical effects of a catalyst inside plasma reactors seem to be mostly clear. These effects include the change in discharge behaviour, electric field enhancement and an enhanced electron energy distribution.^167^ However, the chemical effects of a catalyst inside plasma are not well understood yet. The primary reason is that it is impossible to precisely differentiate the physical and chemical effects when the catalyst is placed inside the discharge zone. The second is the lack of sufficient experimental techniques for a clear-cut understanding of the surface processes at the solid–gas interphase. CH_3_OH selectivity in the presence of various metal species (Pt, Fe_2_O_3_, Cu, Zn, CeO_2_) on dielectric supports (ceramic, Al_2_O_3_) in plasma is presented. However, a proper explanation on the chemical effects of the catalyst is not properly explained.^107,228^

It was already mentioned that the covalent nature of the chemical bonds is the major problem for getting CH_4_ coordinated to an active transition metal. However, this is not the case for CO_2_. As CO_2_ is a Lewis acid, its ability to react or coordinate with various basic compounds has been utilized for CO_2_ capture, fixation and activation.^229^ A representative example can also be seen in the case of plasma assisted conversion of CH_4_ with CO_2_ in a DBD reactor. Among Ni catalysts supported on various compounds (γ-Al_2_O_3_, MgO, SiO_2_, and TiO_2_), CO_2_ conversion rate decreased in the order Ni/γ-Al_2_O_3_ > Ni/MgO > Ni/SiO_2_ > Ni/TiO_2_ whereas plasma only conversion yielded an even lower conversion rate. One of the expected reasons for a higher CO_2_ conversion rate in the case of Ni/γ-Al_2_O_3_ was due to a greater number of strong basic sites on the catalyst surface, which increased the residence time of the molecule in the discharge zone and increased the extend of interaction with the reactive species. Furthermore, the catalyst showed a higher selectivity towards syngas and C-3 to C-4 hydrocarbons as well as a lower carbon deposition.^160^

For better active metal selection strategy for CH_4_ and CO_2_ valorisation, thorough understanding of the intermediate transition states and reaction pathways is necessary. Recently, Zhao *et al.* reported a density functional theory calculation for the efficient coupling of CH_4_ with CO_2_ over Zn doped Ce catalyst in plasma free atmosphere.^230^ The article nicely presented the formation of Zn–CH_3_ bonds by dissociative chemisorption of CH_4_. Later, the insertion of CO_2_ yielded a three centred metal–acetate transition state, which can regenerate the catalyst by means of CH_3_COOH elimination. Even though the study was reported for a plasma free catalytic conversion, the extension of such models into hybrid plasma systems would enable the experimentalist to choose a better catalyst for hybrid plasma catalytic systems.

In the case of plasma catalytic systems, such modelling can pose a significant challenge since plasma is a complex mixture of numerous excited species. Furthermore, while taking into account the influence of various plasma parameters and the influence of the catalyst on the properties of plasma, such modelling becomes even more complicated. Moreover, the influence of the catalyst on the properties of plasma is still unclear. Before concluding this section, it's worth quoting a question put forward by Prof. J. C. Whitehead, in one of his recent articles:^194^ ‘Will it be possible to design a catalyst that can be activated by plasma but is inactive thermally?’ Due to limitations in the fundamental understanding of the basic mechanisms, to achieve such a milestone would be a great challenge for the plasma community.

## 9. Plasma assisted catalytic conversion of CH_4_ and CO_2_ into valuable chemicals

The field of plasma assisted catalytic conversion of CH_4_ and CO_2_ has been given great attention in recent times. The results from recent publications are summarised in Table 4. One of the accepted approaches for CO_2_ utilization is to reduce it with H_2_ inside a catalyst embedded plasma reactor, producing liquid fuels such as CH_3_OH.^206^ By introducing a commercial CuO/ZnO/Al_2_O_3_ catalyst inside the discharge zone, CH_3_OH yield increased up to 10 times compared to the one obtained in catalyst free discharge at a gas temperature of 100 °C. Furthermore, the selectivity increased up to 20%. One of the addressed problems in CO_2_ reduction was a very low CH_3_OH selectivity (<1% in the presented example) and a large degree of H_2_ consumption during the process. To overcome the above mentioned issues of H_2_ consumption and low liquid product selectivity, widely accepted method is the CO_2_ reforming by CH_4_, which allows the large scale utilization of both gases. Furthermore, readily available hydrocarbons like C_2_H_6_ can also be used as reagents for CO_2_ reduction.

**Table 4:** Summary of CH_4_ and CO_2_ valorisation in various hybrid plasma catalytic reactors

Feeding gas ratio	Reactor configuration	Conversion (CO_2_/HCs)	T (°C)	Packing material	Value added products and selectivity			Reference
CO_2_ + H_2_ (1 : 3)	DBD; discharge gap 1 mm,	14%/—	100	CuO/ZnO/Al_2_O_3_	CH_3_OH (7–10%)			249
CO_2_ + C_2_H_6_	DBD	NA/100%	RT	VO_*x*_/Al_2_O_3_	HCHO (11.4%)			250
CO_2_ + CH_4_ (1 : 3)	DBD, 500W				C-2	C-3		239
		NA		Fleece	11.3	8.7		
		NA		NaA	13	10.3		
		NA		NaY	12.3	10		
		NA		HY	15.2	11		
CH_4_ + CO_2_ + Heat flow of 14 + 1+ 65 mL min^−1^	DBD, *V* = 18 kV, frequency: 300 Hz				C-2	C-3	C-4	241
		23.3/19.5	RT	La_2_O_3_/γ-Al_2_O_3_	42.9	12.4	5.6	
		32.0/20.9	200	La_2_O_3_/γ-Al_2_O_3_	41.2	15.0	8.7	
		56.1/21.4	400	La_2_O_3_/γ-Al_2_O_3_	39.5	11.7	5.3	
CO_2_ + CH_4_ (1 : 2)	Corona discharge with hollow Cu ground electrode; power 30 W, feed flow rate 25 mL min^−1^				C-2			240
		16.7/43.4	RT	γ-Al_2_O_3_	30.6			
		22.1/24.5	RT	5% La_2_O/γ-Al_2_O_3_	70.6			
		22/23.8	RT	0.1% Pd–5% La_2_O/γ-Al_2_O_3_	70.4			
CH_4_ + air (1 : 1)	*P* = 140 W; discharge frequency, 7 kHz; DBD discharge gap = 2.5 mm				CH_3_OH			251
		—/25–26%	No effect	Plasma only	∼7.6%			
			150	Ceramic pellet (CP)^a^	>8%			
		—/25–26%	150	Pt/CP^a^	∼9%			
		—/25–26%	150	Fe_2_O_3_/CP^a^	10.66%			
		—/25–26%	150	CeO_2_/CP^a^	>8.5%			
CH_4_ + air (1 : 1)	DBD discharge gap = 2.5 mm, feed flow rate = 300 sccm				CH_3_OH			228
		—/24.5–25.5%	150	CP^a^	∼8.5%			
		—/24.5–25.5%	150	CuO/CP^a^	9%			
		—/24.5–25.5%	150	Fe_2_O_3_/CP^a^	10.1%			
		—/24.5–25.5%	150	Fe_2_O_3_–CuO/CP^a^	11.3%			
CH_4_ + air	*P* = 61 W; discharge frequency,7 kHz; DBD discharge				CH_3_OH			252
		—/35%	150	CuO/γ-Al_2_O_3_	2.5%			
		—/36%	150	Mo–CuO/γ-Al_2_O_3_	3.5%			

a Catalyst in the afterglow.

In a very recent report, plasma assisted catalytic conversion of CO_2_ by C_2_H_6_ is achieved. A V^5+^/Al_2_O_3_ catalyst, which is known to be one of the most efficient catalysts for oxidative dehydrogenation of C_2_H_6_, was used for the reaction.^231^ The catalyst was embedded in the discharge zone along with glass balls and BaTiO_3_. Major reaction by-products obtained include H_2_, CO, CH_4_, C_3_H_6_ and HCHO with a 100% C_2_H_6_ conversion and high HCHO selectivity (11.4%). The improvement in selectivity was attributed to the synergistic effects of active vanadium catalyst, ferroelectric BaTiO_3_ and plasma activation. The process allows a successful utilization of CO_2_ for the production of HCHO, a necessary chemical in industry used for wood processing, textile manufacturing and the production of formaldehyde resins, fertilizers, chelating agents and polyhydric alcohols.^232^

The ability of Cu to adsorb CO_2_ as COO^−^ species on the surface, which will then reduce to a crucial intermediate HCOO^−^, is an important advantage of Cu the plasma catalytic conversion CO_2_ into alcohols. This was validated by Zhao *et al.* in the selective synthesis of ethanol from CO_2_ and water vapour with the assistance of commercially available Cu/ZnO/Al_2_O_3_ catalyst packed in a negative corona reactor. It is assumed that the active Cu species on the surface are partially oxidised in the plasma, which provides better selectivity towards ethanol compared to other competing products such as methanol.^233^

Oxidative CH_4_ coupling of CH_4_ with CO_2_ in hybrid plasma catalytic reactors significantly increases the CH_4_ conversion along with the inhibition of carbon deposits. During such reactions, the major product obtained in many reports is syngas.^234–236^ In addition to that, a large number of gaseous hydrocarbons (C_2_H_6_, C_2_H_4_, *etc.*), liquid hydrocarbons (pentane), and organic oxygenates could be also produced. It was presented that by introducing CO_2_ in the feeding gas, the conversion of CH_4_ over catalysts inside a DBD can be increased. The mechanism behind this is well explained in literature.^234^ Dissociation of CO_2_ can yield atomic oxygen species (*e.g.* O(^1^D)), which can easily pick up a hydrogen atom from CH_4_ to generate CH_3_ radicals. These hydrocarbon radicals will further react with other hydrocarbon radicals, atomic oxygen or OH radicals to yield higher hydrocarbons or liquid oxygenates.

It is well known that one can change the properties of plasma by changing the electrode material used for its generation.^237^ Using this principle, optimal plasma-catalyst synergy has been reached for the reduction of CO_2_ with CH_4_ inside DBD with various metallic electrodes.^238^ When the electrode material was changed from steel to Ni or Cu, the selectivity towards carboxylic acids were almost doubled. In this study, the authors gave much attention to the basic understanding of plasma-catalyst interactions rather than achieving a better product yield. Such research should be encouraged to cover the gap between plasma assisted conversion and the underlying mechanisms associated with it.

Zhang *et al.* compared the effects of various zeolite packings inside DBD on the selectivity towards higher hydrocarbons and liquid fuels.^239^ Incorporation of zeolite HY in the discharge zone significantly reduced both CH_4_ and CO_2_ conversion rates whereas selectivity towards C-4 hydrocarbons reached above 50%. On the other hand, the selectivity towards C-4 hydrocarbons was significantly lower in zeolite NaY packed reactors. The selectivity towards C-4 hydrocarbons has been found to decrease depending on the packing material in the order, zeolite HY > zeolite NaA > zeolite NaY > fleece. This study further supported the previous statement that a very small change in the chemical properties of the catalyst can significantly influence the reaction pathway or resulting products in a hybrid plasma reactor. The explanation for such observations is still a puzzle for the plasma catalysis community.

One of the most convenient ways to achieve higher hydrocarbon yield is to increase the discharge power. However, the yield of liquid products such as CH_3_OH or lower hydrocarbons tends to decrease with an increase in discharge power. It is assumed that lower hydrocarbons get activated and are converted into higher ones at elevated powers. However, these products are being degraded by plasma (and the elevated temperature at higher power) as well, so after some point, the increased plasma power will negatively affect the amounts of higher hydrocarbons in the product stream, and products such as H_2_, coke, and CO will be prevalent. It should be noted that the properties of plasma vary significantly with the changes in discharge power and thus the reaction pathways in plasma catalysis are completely modified. Thus it is very difficult to make a general conclusion on such influences of discharge parameters.

In CH_4_ conversion with CO_2_, sufficient amount of CO_2_ is necessary to provide an oxidizing atmosphere, which can reduce the carbon deposition and following catalyst deactivation. Zhang *et al.* presented the effect of CO_2_ concentration on product selectivity during C-2 hydrocarbon formation inside a hybrid pulse corona La_2_O_3_/γ-Al_2_O_3_ catalyst reactor.^240^ As CO_2_ content in the feed was increased by a factor of 4, C_2_H_2_ selectivity decreased by about 35% along with a significant increase in the formation of C_2_H_6_ and C_2_H_4_ (5 to 26% and 7 to 16% respectively). By increasing the CO_2_ concentration in the feed gas, it is possible to increase the CH_4_ conversion. However, for higher concentrations of CO_2_, the selectivity towards higher hydrocarbons or alcohols tends to decrease with a simultaneous increase in the selectivity towards CO or CO_2_ in the outlet. Furthermore, the influence of Pd doping on La_2_O_3_/γ-Al_2_O_3_ in the plasma catalytic conversion of CH_4_ over CO_2_ was studied. It was revealed that traces of Pd on La_2_O_3_/γ-Al_2_O_3_ increased the C-2 selectivity up to 70% and along with a high C_2_H_4_ (about 65%) in the product mixture. Whereas in the case of corresponding La_2_O_3_/γ-Al_2_O_3_ without Pd yielded very high C_2_H_2_ (76%) with C_2_H_4_ yield below 12%. The results revealed the efficiency of Pd–La_2_O_3_/γ-Al_2_O_3_ catalyst for C_2_H_2_ hydrogenation under pulse corona discharge. On the other hand, the yield towards C-2 HCs was lower inside La_2_O_3_/γ-Al_2_O_3_ hybrid DBD reactor at a discharge frequency of 300 Hz. However, inside the hybrid DBD reactor, there was significantly higher selectivity of C-3 and C-4 HCs (12.4 and 5.6% respectively) at room temperatures.^241^ Such differences in the yield and selectivity might be attributed to the difference in the plasma-catalyst interactions arose due to the distinct properties of generated plasmas in different reactor configurations. Another approach for the valorisation of CH_4_ is its controlled oxidation with atmospheric air or O_2_ due to cost effectiveness. In a related study, the effects of various catalysts deposited on ceramic particles (CP) in a two stage hybrid plasma catalyst reactor revealed that CH_3_OH formation was occurring in two stages.^107^ In the first stage, CH_*x*_ radicals formed as a result of CH_4_ dissociation and then reacted with OH radicals and a sufficient number of atomic H to form CH_3_OH. Syngas was also formed as a by-product. In the second stage, this synthesis gas was converted on the active catalyst surface to yield CH_3_OH. At a moderate temperature of 150 °C, the CH_3_OH selectivity for various catalysts decreased in the order Fe_2_O_3_/CP > Pt/CP > CeO_2_/CP > CP > plasma only. The incorporation of a Fe_2_O_3_ catalyst increased the CH_3_OH selectivity by up to 50% compared to that of plasma only process.

In thermal catalysis, Cu is widely used as a promoter to improve the activity and selectivity of Fe_2_O_3_ catalysts towards alcohols.^242^ The same catalyst selection strategy has been applied in a two stage plasma catalytic conversion process.^228^ The comparison of catalyst performance in plasma revealed that CH_3_OH selectivity was 10.5% lower when Fe_2_O_3_/CP was used instead of the CuO modified catalyst. The improvement in the activity of the catalyst was ascribed to a reduced electron density around Fe^3+^ as well as a reduction in oxygen vacancy concentration after CuO doping. Furthermore, it was revealed that the CuO promoter didn't have much influence on CH_4_ conversion, but merely improved CH_3_OH selectivity. Similar effects of Mo doping of a CuO/Al_2_O_3_ catalyst on the selectivity towards CH_3_OH formation were also reported.^207^ The mechanism of CH_3_OH production is speculated as follows, where the influence of the oxygen vacancies is considered.

1 CH_4_ + e → CH_3_ + H + e

2 O_2_ + e → O + O + e

3 H + O + CH_3_ + 2M–O → M–OCH_3_ + M–OH

4 M–OCH_3_ + M–OH + O → CH_3_OH + 2M–O

5 V_0_ + O = C → V_0_⋯O^2−^⋯C^2+^

6 V_0_⋯O^2−^⋯C^2+^ + 2H_2_ → V_0_ + CH_3_OH

where V_0_ and M–O is the oxygen vacancy and the metal oxides of Mo–CuO/Al_2_O_3_ sample, respectively.^207^

Both single stage and two stage plasma catalytic systems are well known and widely exploited for various applications including molecular abatement, CH_4_ or CO_2_ activation, air purification and many more. However, the studies have to be extended into liquid fuel synthesis from CH_4_ to delve more deeply into plasma-catalyst interactions and resulting molecular conversion and product selectivity.

A study presented on the partial oxidation of CH_4_ with air over a Fe_2_O_3_–CuO/γ-Al_2_O_3_ catalyst using DBD compared the effects of various catalyst packing strategies.^243^ Inside the single stage plasma reactor, CH_4_ conversion rate was much higher and the yielded products indicated higher extension of total CH_4_ oxidation in a reactor packed solely with Al_2_O_3_. On the other hand, with a Fe_2_O_3_–CuO/γ-Al_2_O_3_ catalyst, CH_3_OH yield was 68% higher than that of pure Al_2_O_3_ at 200 °C. Whilst in the case of a two stage plasma reactor, the maximum CH_3_OH yield achieved with the Fe_2_O_3_–CuO/γ-Al_2_O_3_ catalyst was only 21% higher at a temperature of 150 °C. By introducing the catalyst in the plasma zone, it was possible to reduce the unwanted ozone emission in the outlet.^19,244^ However, the catalyst embedded in the discharge zone was more prone to carbon deposition and consequent deactivation.^243^

Some researchers currently focus on the efficiency of various plasma catalytic systems to oxidize CH_4_ into liquid fuels in the presence of oxides of nitrogen like N_2_O. Even though such processes are promising for new advance in the field, the conversion of CH_4_ is always preferred using cheap oxidants including O_2_, air or CO_2_ since most of the CH_4_ depositions are in remote regions and the usage of reagents which are readily available at the location is necessary.

## 10. Economic viability and industrialization of plasma assisted conversion

The energy efficiency, economic viability and ease of scale up are some of the important aspects of any process evaluation. One of the approaches would be to calculate the energy needed for the formation of unit mole of a particular compound. Some of the lowest electricity costs of methanol, produced by plasma oxidation of methane, that we found through our literature survey, were 69 kW h kg^−1^,^117^ 87.7 kW h kg^−1^,^116^ 142.6 kW h kg^−1^ (ref. 114) and 201.9 kW h kg^−1^.^108^ Among these results, the cheapest price of electricity per liter of methanol produced would be roughly 6.5$ per l, assuming an approximate electricity price of 12 cents per kW per h. This price is obviously not taking into account the price of the feedstock, other operational costs and transport. For comparison, current market methanol production costs are on the approximate level of 0.1–0.3$ per l.^245^

**Fig. 9:**
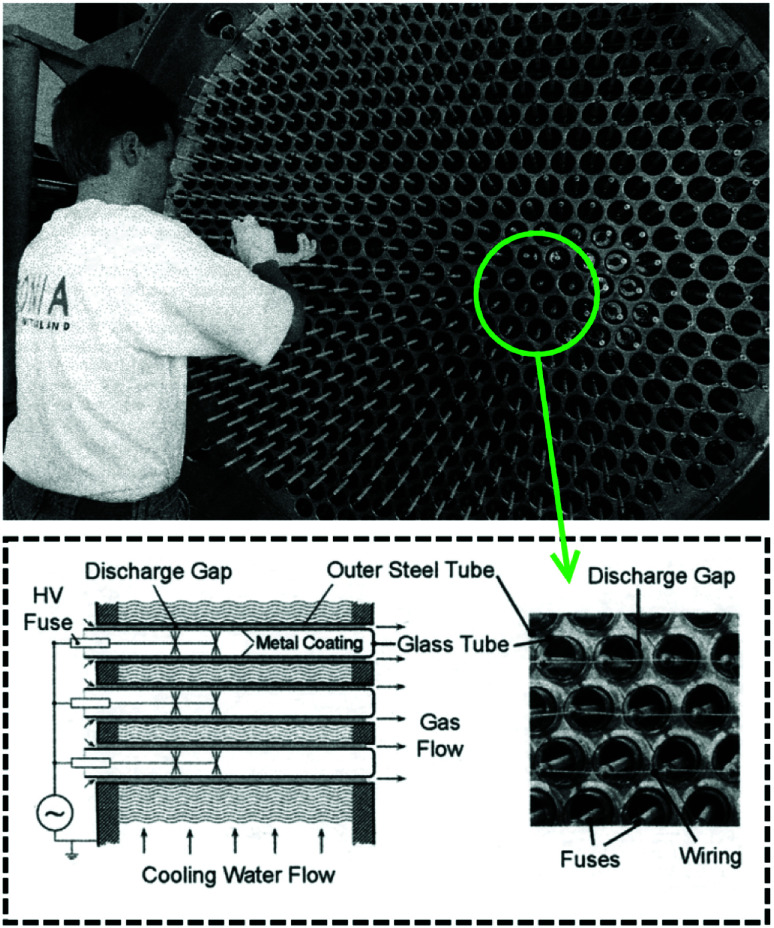
Assembly of smaller reactor bundles for the industrial scale plasma scale processing (top) and the enlarged view of smaller reactor (bottom). Reproduced from ref. 246 with permission from Springer Nature, copyright 2003.

As the research is done on laboratory-scale setups, the direct comparison with industrially produced methanol might not be the fairest, but it can give a very rough idea on the feasibility of the process. For smaller scale production, the costs associated with a plasma reactor may be significantly lower than that of a two-step steam-reforming to methanol synthesis plant. Compared to the thermal catalysis, fast turn on/off time and room temperature operation are significant advantageous of plasma assisted conversion processes as well, which can reduce the energy consumption to larger extend. Nevertheless, one can question the feasibility of plasma catalysis as the process is operated at atmospheric pressure with limited gas inlet flows. Such a drawback can be easily overcome by utilizing the parallel reactor concept for DBD as introduced by Kogelschatz for large scale ozone synthesis.^246^ The industrial scale reactor thus constructed consists of bundles of small scale reactors arranged in parallel, as presented in Fig. 9.^246^ Such reactors allow operations at higher gas inlet flows, incorporation of the catalyst and higher energy input.

The major advantages of plasma operation are attributed to high reactivity and the possibility of low temperature operation. This is beneficial for surpassing certain thermodynamic limitations for the thermal process in cases such as production of organic oxygenates in a single-step process. However, it does not mean that the energy cost of the operation is lower, as plasma can be similarly energy intensive as heating the gas to a high temperature. Therefore, to determine the industrial viability of the process, the energy cost has to be given great attention.

## 11. Conclusions, challenges and future outlook

In this review, we presented an overview of both plasma and plasma assisted catalytic conversion of CH_4_ and CO_2_ into valuable chemicals, which are either liquids or gases with much lower energy for liquefaction compared to CH_4_. The inspiration behind this is based on large scale exploitation and easy storage of widely available natural gas resources with an additional benefit of carbon cycle balance and consequent control of greenhouse effects due to CO_2_ and CH_4_ emission.

The articles presented in this review evidently support the future potential of plasma catalysis for efficient valorisation of greenhouse gases into liquids and other useful fuels on a larger scale. Plasma assisted conversion has multiple benefits such as fast switch on and off times, low temperature operation and reduced coking. Furthermore, synergistic effects inside plasma hybrid catalyst reactors are promising for the improvement of product selectivity and energy efficiency and could help to upgrade conventional thermal catalysis.

However, both the plasma and the catalytic community are still facing numerous challenges regarding the aforementioned technology. One is the over oxidation of the liquid products formed on the catalyst bed due to their high retention times. Extremely high reactivity of the plasma reactive species and micro discharges in the catalyst pores can facilitate this unwanted degradation. Thus it is very important to fabricate suitable catalysts that would enable sufficient micro discharges to enhance CO_2_ and CH_4_ conversions and allow easy diffusion of the desired products from the catalyst bed.

As mentioned before, low temperature operation is one of the key advantages of plasma hybrid catalyst reactors. Even though catalysts operate efficiently at lower temperatures (∼100 °C) when plasma is present, the primary challenge is to build up suitable plasma-catalyst systems that operate efficiently even at room temperatures. This would provide exceptionally good energy efficiency, one of the most important criteria for industrial scale production.

This issue can be overwhelmed by achieving a more precise understanding of the underlying mechanisms and by developing an advanced catalyst selection strategy. As mentioned before, the most commonly accepted strategy for catalyst selection for plasma hybrid reactors is to pick the ones which are efficient for conventional catalytic conversion. A better catalyst selection strategy can be achieved only by combining advanced level simulation on plasma, catalysis and plasma–surface interactions and validate them with dedicated experiments.

Furthermore, scrutiny on the interaction of various excited states (electronically and vibrationally) with the catalyst surface and their prominent role in the product formation is another challenge. Indeed, a development in experimental techniques that allow precise monitoring of the reactions at the plasma-catalyst interface is necessary. Overall, a much deeper fundamental understanding of the process is required in order to bring plasma catalysis closer to the industry, and focused research with strong interdisciplinary bonds is needed to achieve it.

## Conflicts of interest

There are no conflicts to declare.

## Supplementary Material
